# Breaking (musical) boundaries by investigating brain dynamics of event segmentation during real-life music-listening

**DOI:** 10.1073/pnas.2319459121

**Published:** 2024-08-26

**Authors:** Iballa Burunat, Daniel J. Levitin, Petri Toiviainen

**Affiliations:** ^a^Centre of Excellence in Music, Mind, Body and Brain, Department of Music, Arts and Culture Studies, University of Jyväskylä, Jyväskylä 40014, Finland; ^b^School of Social Sciences, Minerva University, San Francisco, CA 94103; ^c^Department of Psychology, McGill University, Montreal, QC H3A 1G1, Canada

**Keywords:** boundary perception, event segmentation, musicians, naturalistic, functional MRI (fMRI)

## Abstract

The perception of musical phrase boundaries is a critical aspect of human musical experience: It allows us to organize, understand, derive pleasure from, and remember music. Identifying boundaries is a prerequisite for segmenting music into meaningful chunks, facilitating efficient processing and storage while providing an enjoyable, fulfilling listening experience through the anticipation of upcoming musical events. Expanding on Sridharan et al.’s [*Neuron* **55**, 521–532 (2007)] work on coarse musical boundaries between symphonic movements, we examined finer-grained boundaries. We measured the fMRI responses of 18 musicians and 18 nonmusicians during music listening. Using general linear model, independent component analysis, and Granger causality, we observed heightened auditory integration in anticipation to musical boundaries, and an extensive decrease within the fronto-temporal-parietal network during and immediately following boundaries. Notably, responses were modulated by musicianship. Findings uncover the intricate interplay between musical structure, expertise, and cognitive processing, advancing our knowledge of how the brain makes sense of music.

## Significance

Understanding how we perceive musical boundaries is crucial for unraveling the intricate processes behind our musical experiences, such as our ability to find enjoyment and meaning in music. Imagine listening to music without discernible segments—its continuous stream would lack structure and become overwhelming or rather dull. By analyzing how the brain reacts to musical phrase transitions in musicians and nonmusicians, we fill a critical gap in music perception and cognition. We noted brain activity changes during boundary transitions with discernible modulations based on musicianship. This emphasizes the impact of expertise on refining our neural processing and underscores a fundamental language-like system for temporal segmentation in the brain, with broader implications for auditory scene analysis beyond the domain of music.

Imagine listening to music without the ability to mentally parse what you hear. The music would seem like a never-ending stream of random sounds, completely lacking in any structure or meaning. The experience would likely be overwhelming and difficult to make sense of, like looking perpetually through a kaleidoscope. Although music unfolds over time and a single composition can last for over an hour, the notes themselves adhere to grouping principles and comprise discrete events or musical segments. Without the ability to detect the boundaries between musical events, all structure and meaning of the composition would be lost; the listener would miss out on the many ways in which different musical elements interact to create the whole. Critically, the confused listener would miss out on the emotional impact and significance of the music.

The ability to perceive boundaries not only allows us to understand and appreciate music, but is also fundamental to our most basic everyday functioning. Recognizing boundaries allows us to distinguish information from the continuous flow of sensory signals we receive (1). Boundary detection is a prerequisite for feature extraction, a preparatory operation for object identification and subsequent memory encoding: It is only by segmenting and organizing the world into smaller meaningful units and relating them to each other that we can make sense of it.

When events are organized into hierarchical structures, categorized into different levels of detail, and occurring at various time intervals, boundary detection represents a core process that sustains our perception, attention, memory, and decision-making. Parsing ongoing information into events is tied to updating working memory, accessing long-term memory, and learning new skills (2). Event segmentation may have resulted from an adaptive mechanism that integrates recent information to improve predictions about the near future (1).

In humans, musical experience relies heavily on the recognition of phrase and event boundaries, which are crucial for comprehending musical structure and meaning. This operation is essential for memory encoding, as events must be segmented into a beginning and end for storage (3).

Music is a dynamic process of information that unfolds over time, with auditory elements such as melody, rhythm, timbre, loudness, and harmony interacting to create a hierarchical structure. As we listen, our brains process this structure by extracting low-level and high-level features, identifying patterns, and predicting what will happen next (4–7). This process engages our attention and cognitive resources because our working memory is updated continuously as we make sense of the evolving flow of musical information.

A complex flow of undifferentiated information from the environment impinges on our eardrums, and from this, higher cognitive operations serve to differentiate, organize, and categorize that information flow, in part through the perceptual-cognitive process of boundary identification. The neurobiological underpinnings of this process are not well understood. Few neuroimaging studies that might offer answers fall short in providing comprehensive insights as the stimuli used in such methods are typically oversimplified and fail to mirror the intricacy of information processed by the brain in more diverse and realistic environments.

**Fig. 1.**
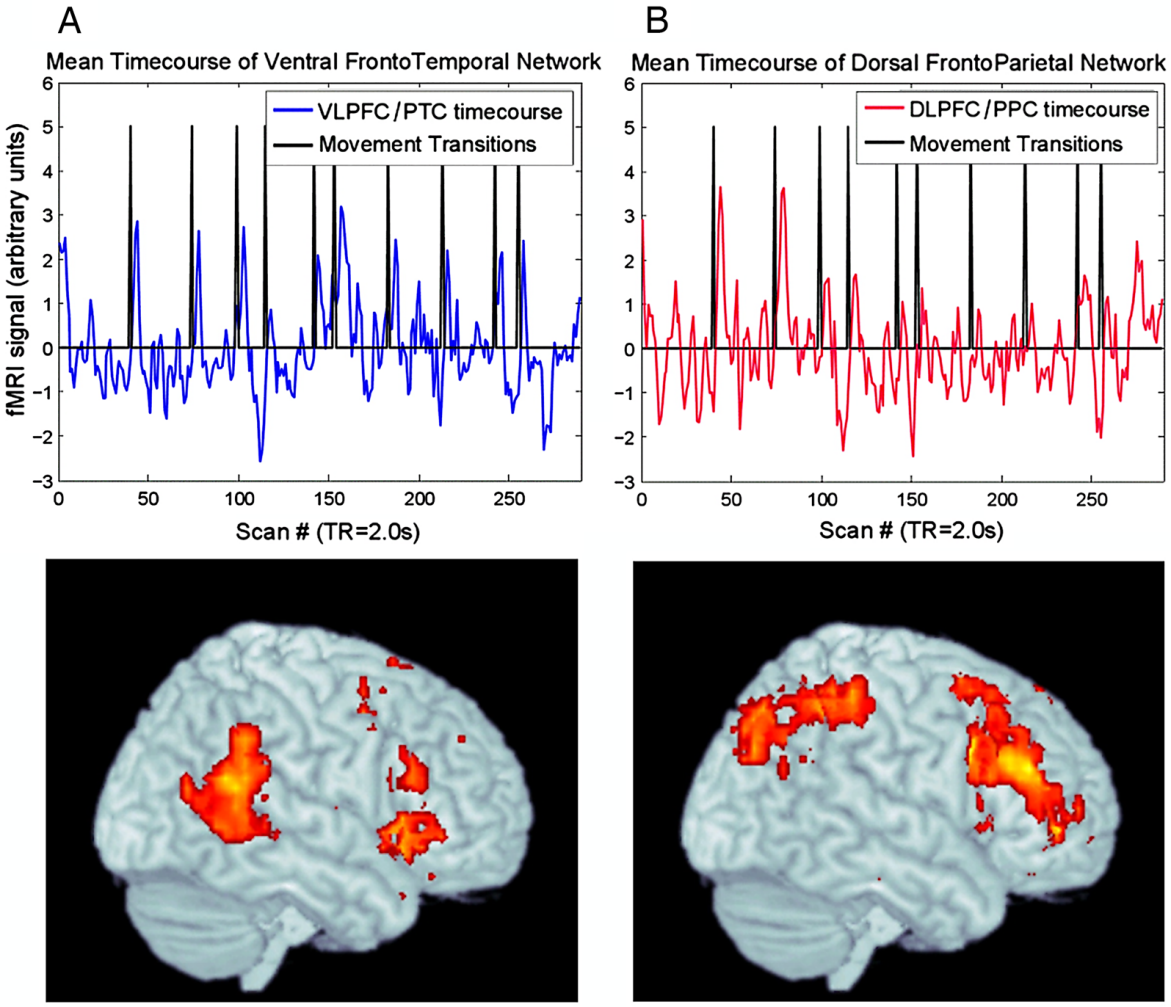
Results of Sridharan et al. (8). An early network (*A*) including the ventrolateral prefrontal cortex (VLPFC) and posterior temporal cortex (PTC) responded to movement boundaries followed by a (*B*) network including the dorsolateral prefrontal cortex (DLPFC) and posterior parietal cortex (PPC). Reproduced, with permission, from Sridharan et al. (8).

The sole neuroimaging study, to our knowledge, that examined brain changes occurring at boundary transitions using music that simulates real-world music listening, was conducted by Sridharan et al. (8). They tracked the fMRI activity of musically untrained listeners during symphonic movement transitions, that is, coarse-grained boundaries (musical excerpts of symphonies by William Boyce). Findings revealed two relevant brain networks: a ventral fronto-temporal network relevant to detecting salient events and a dorsal fronto-parietal network relevant to information maintenance and working memory (Fig. 1). This dorsal/ventral distinction suggests different networks for voluntary versus involuntary attentional sets, as have previously been identified in visual perception (9).

Within an ecologically valid musical context, our current study aimed to explore the brain dynamics underlying fine-grained segment boundaries perception in both musicians and nonmusicians, as they listened to music spanning different genres. In doing so, our research seeks to bridge a substantial gap in the music cognition and neuroscience field, namely, the nuanced exploration of fine-grained musical boundaries, modeled as a continuous variable obtained from a real-time listening task, while extending this investigation to encompass different musical genres. Equally, our study seeks to address the lesser-explored influence of listeners’ musical training on processing fine-grained musical boundaries within this paradigm.

Musicians often exhibit a noticeable pattern of leftward cerebral activity in the auditory association areas and prefrontal cortex compared to nonmusicians (10, 11). This distinctive neural activity is believed to be linked to the functional reorganization that occurs as a result of long-term training. Additionally, musicians have also shown larger blood-oxygen-level-dependent (BOLD) responses in attention and cognitive control-related networks during working memory tasks involving musical sounds, distinguishing them from nonmusicians (12). Moreover, musicians demonstrate overall larger responses to music compared to controls (13, 14). Therefore, the inclusion of musicians in our study provides a unique opportunity to elucidate how musical expertise impacts the brain processing of musical boundaries.

Following Sridharan et al. (8), we used fMRI and an experimental design that simulated real-world music listening to analyze brain activity associated with transient changes, i.e., segmentation boundaries at a finer-grained hierarchical scale (phrasal segmentation) than those investigated in their study. We obtained these nuanced, fine-grained segment boundaries from a real-time music-listening task by identifying the temporal locations in the musical stimuli where participants agreed on the presence of boundary markers. This was achieved through the use of kernel density estimation (KDE) analysis, which yielded a continuous measure that captured the degree of consensus across participant groups. This approach allowed us to capture the subtle nuances of boundary perception and identify the varying degrees of salience associated with each boundary, while also providing an objective measure of perceptual agreement. This way we derived a segmentation variable obtained using an ecologically valid approach. Notably, our modeling approach differs fundamentally from that of Sridharan et al. (8), in two key aspects. First, while Sridharan et al. utilized notational data, our study employs perceptual data derived from a real-time listening task. Second, Sridharan et al. (8) used binary variables, categorizing each time point as either a boundary or not, whereas our approach employs continuous values assigned to each time point, representing the salience of a given time point being identified as a boundary. This distinction underscores our endeavor to capture the nuanced and probabilistic nature of boundary perception within a dynamic musical context.

To generalize results and mitigate the influence of individual musical preferences, participants listened to three distinct musical pieces of different genres, enabling us to generalize our findings, a dimension not explored in the work of Sridharan et al. (8), who exclusively examined the English Baroque symphonic work of a single composer. Using three contrasting genres of music not only helps to counteract potential biases associated with specific musical styles but also leverages the high variability inherent in our chosen stimuli. This increased variability improves the chances of capturing perceptual changes in brain responses, a crucial consideration given the reliance of most time series statistical methods on covariance. Furthermore, an additional aspect of the present experimental design is the inclusion of both musically trained and untrained listeners to test for potential effect of musical training on the brain circuits responsible for the processing of musical boundaries. This musicianship factor was absent in Sridharan et al. (8).

We hypothesized that these fine-grained musical boundaries would elicit distinct fMRI responses at event boundaries that would intersect with those revealed by Sridharan et al. (8)’s study. While we cannot definitively anticipate significant differences in the brain responses between musicians and nonmusicians to musical boundaries, there is a possibility that such differences may exist based on previous research on the effects of musical expertise in the field.

## Results

### GLM Analyses: Hemodynamic Changes during Musical Boundary Transitions but No Differences across Lags.

To obtain a measure of perceptual boundaries (“boundary regressor”), an experiment was conducted to identify transition points in music by having participants (n = 36) mark “instants of significant change” in real time while listening to music (see *SI Appendix*, Supplementary Methods for a detailed description of how we obtained this perceptual measure).

Additionally, to ensure that any impact of amplitude fluctuations was eliminated and did not confound our results, we followed the approach of Sridharan et al. (8) and regressed out the variance explained for by the RMS of the musical stimulus (estimated using the MIRToolbox) (15) from the brain responses prior to conducting analyses. The residual data were then employed for all analyses carried out in the present study. Additionally, we conducted a Pearson’s correlation test which indicated that the boundary regressor did not predict amplitude fluctuations (r = 0.05; *P*-value = 0.18). Thus, we can infer that responses in the residual data were not attributable to amplitude variation in the stimulus.

**Fig. 2.**
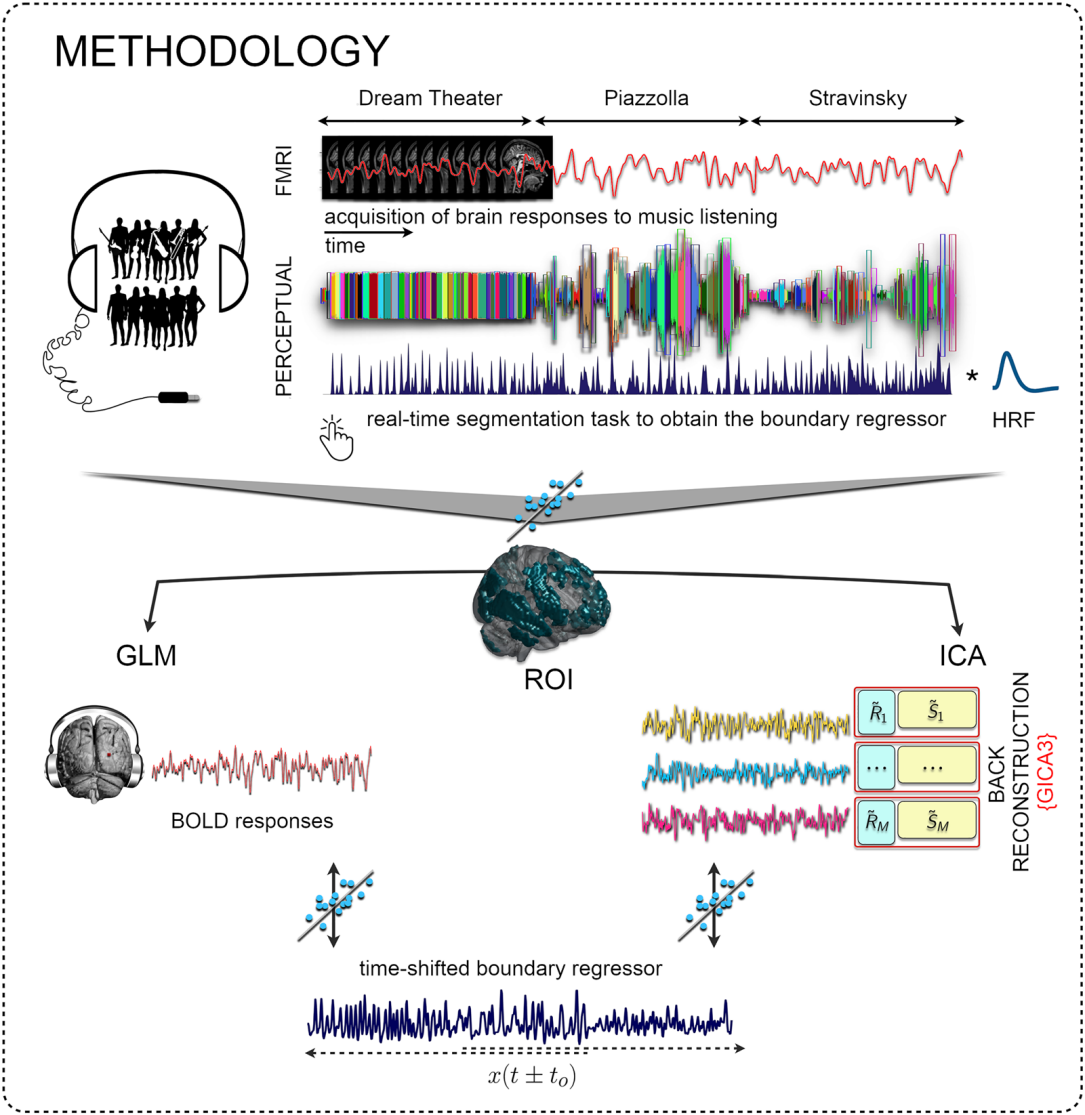
Pipeline of the study.

Using time-shifted versions of the boundary regressor (at −1, 0, and +1 lags; lag = scan interval = 2 s), we performed GLM analyses (refer to Fig. 2 for an illustration of the methods’ pipeline) and observed statistically significant changes in brain activity not only at boundaries but also during the pre- and postboundary transitions across all participants (*P* < 0.001, cluster-wise corrected, FWE = 0.05).

**Fig. 3.**
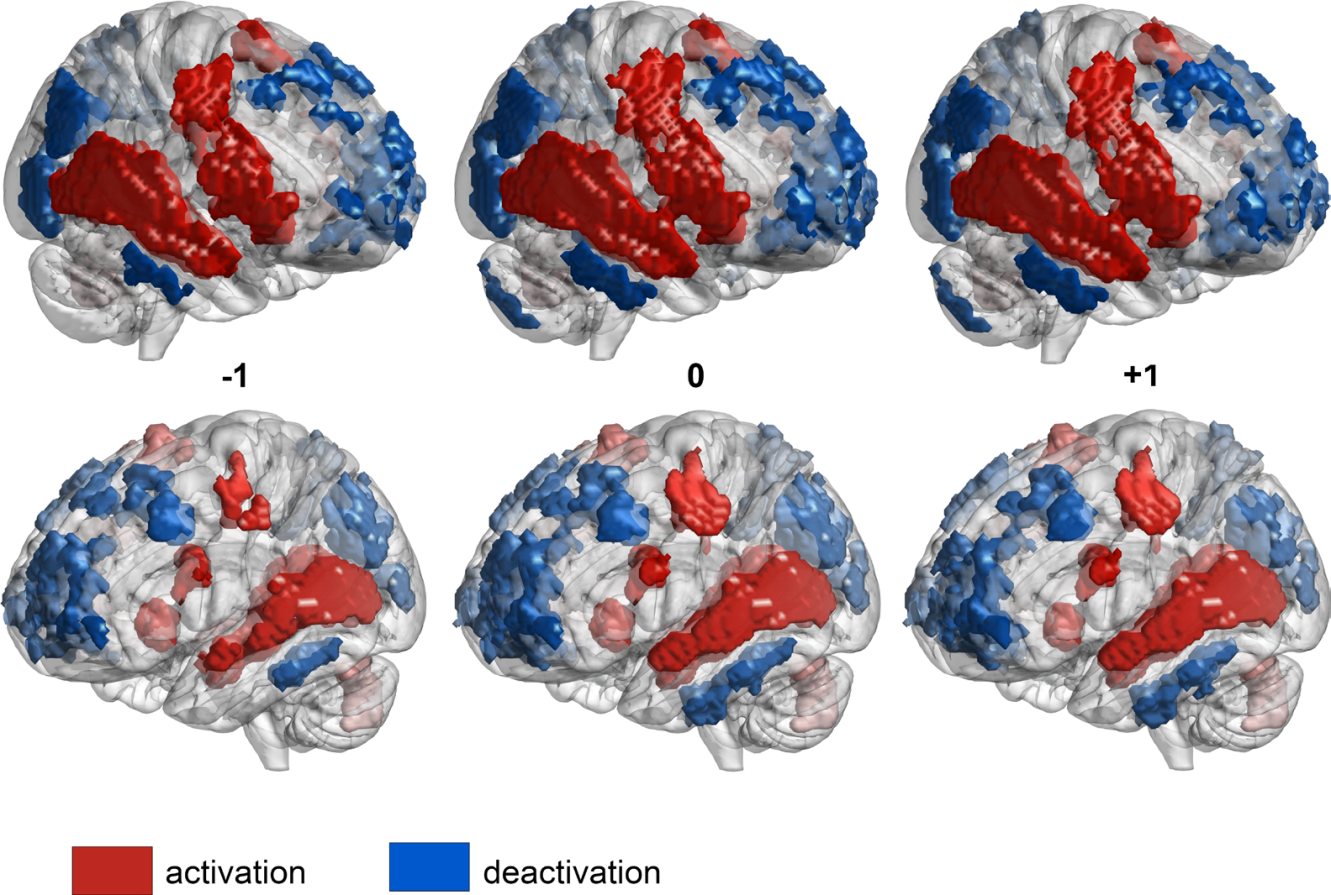
GLM analyses results: hemodynamic changes during musical boundary transitions but no differences across lags. GLM results across lags (before, during, and after boundary transitions; up to 2 s (1 scan = 2 s) before and after the boundary; activations displayed at *P* < 0.001, cluster-wise corrected, FWE = 0.05). Red and blue color codes for activations and deactivations, respectively.

During the boundary transitions, activity increased in fronto‐temporal regions. This encompassed auditory-motor areas with a right-hemispheric asymmetry (primary auditory cortices [BA41/42] and primary motor cortex/supplementary motor areas [BA4/6]), together with the ventrolateral prefrontal cortex (VLPFC, BA44/45/47), also with a rightward bias, and left cerebellum. At the same time, activation decreased in fronto-parietal areas, including the orbitofrontal cortex (OFC, BA10/BA11) and dorsolateral prefrontal cortex (DLPFC, BA8/9/46), notably in the left hemisphere. Likewise, the bilateral posterior parietal cortex (PPC, BA40) exhibited decreased activation. See Fig. 3 and *SI Appendix*, Table S1 for a comprehensive list of brain regions.

**Fig. 4.**
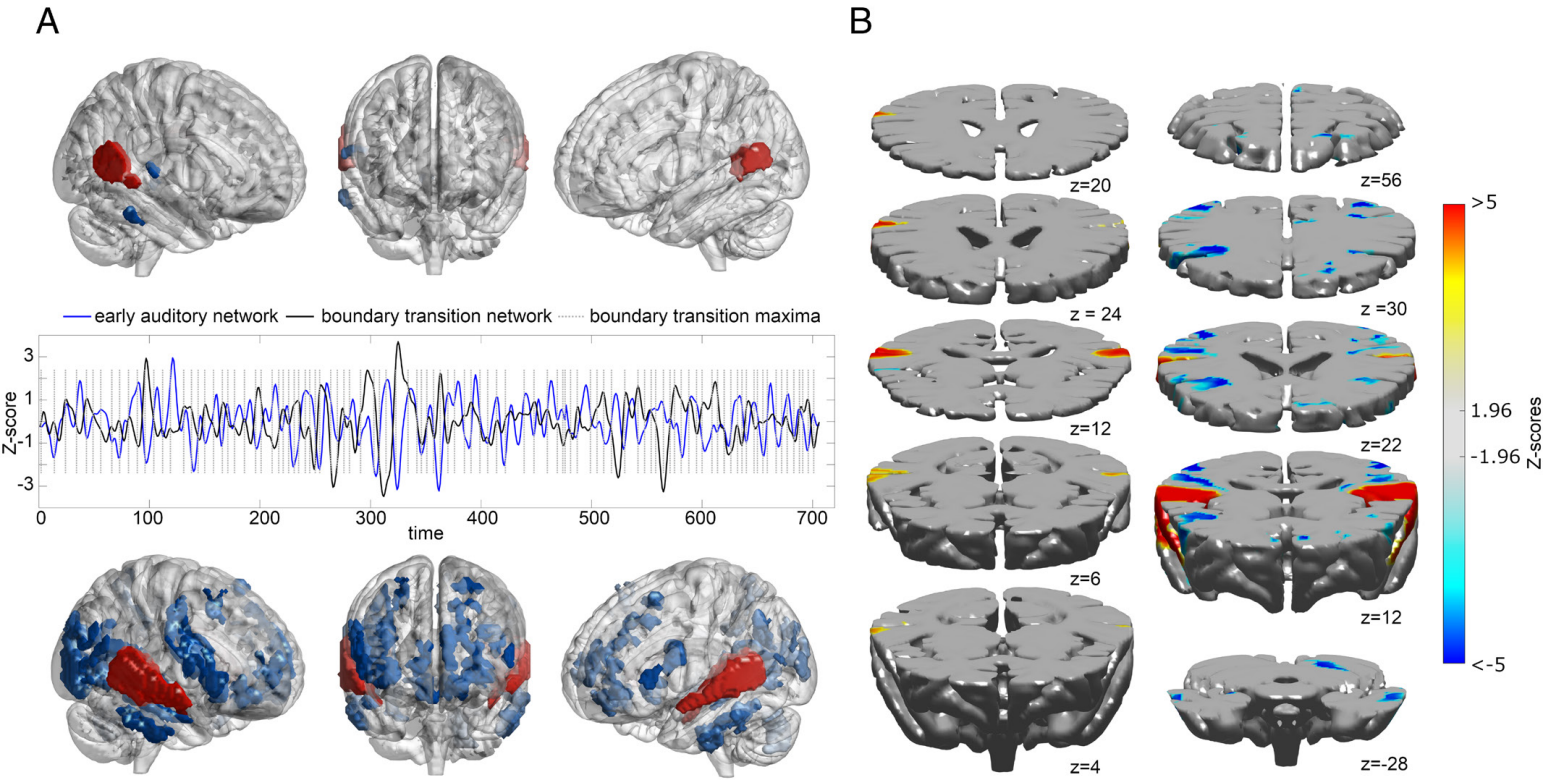
ICA analyses results: distinct networks underpin different phases of boundary transitions. IC functional networks (*A*; *Top* and *Bottom*) with their averaged temporal courses across participants and superimposed boundary transition maxima. *Top*: Early Auditory Network (IC_t=−1_), prior to the transition; *Bottom*: Boundary Transition Network (IC_t=0,1_), engaged during and after the transition. The IC temporal courses consistently tracked boundary transitions (vertical dashed lines) despite the absence of explicit boundary transition models during ICA decomposition. Axial slices (*B*) showing the continuous Z-map for both the Early Auditory (*Left*) and Boundary Transition (*Right*) networks.

However, GLM results did not show regional activation differences across lags. The pattern of brain activity was not statistically different before, during, and after boundary transitions (two-tailed, pairwise Wilcoxon rank sum tests, alpha = 0.05, cluster-wise corrected, FWE = 0.05; see Fig. 4). It is possible that GLM analyses were not sensitive enough to detect significant differences between the lags analyzed.

Refer to *SI Appendix*, *Supplementary Methods*, to see highly consistent results for a nonparametric version of this analysis.

### ICA Analyses: Distinct Networks Underpin Different Phases of Boundary Transitions.

While univariate analyses, like GLM, offer valuable insights into brain activity, they do not provide information on interactions or reciprocal relationships between different brain regions. In contrast, ICA considers all reciprocal relationships between voxels simultaneously as a multivariate approach. Furthermore, ICA analyses avoid a priori assumptions about the shape of the response and aim to extract spatial patterns of brain responses that are statistically independent, together with their associated temporal courses. We applied ICA following the approach described in ref. 16, which avoids making assumptions about an optimal model order. Instead, ICA was computed over a range of model orders that were further examined (see *SI Appendix* for a description). This approach resulted in a more complex and informative pattern than the GLM/univariate analyses. Compared to the univariate GLM approach, sICA demonstrates higher sensitivity in detecting task-related changes in fMRI signal (17). This increased sensitivity arises from a stricter criterion for spatial independence (non-Gaussianity) among IC spatial maps, which helps to distinguish artifacts and physiological fluctuations from the fMRI signal of interest, reducing noise (18).

**Fig. 5.**
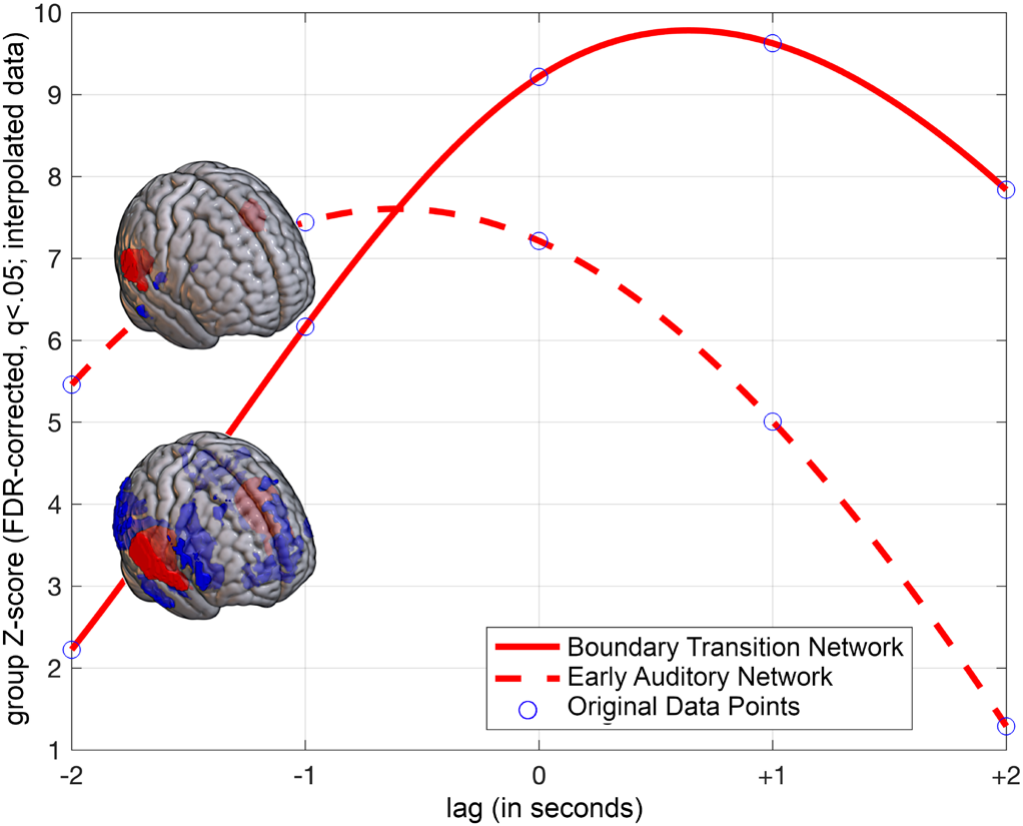
Significance of the IC functional networks as a function of lag; one-sample Wilcoxon Signed Rank Test, N = 36, *P* < 0.001, cluster-wise corrected (FWE = 0.05). The curve in the figure has been interpolated using a cubic spline algorithm for visualization purposes.

ICA analyses revealed two distinct and statistically significant functional networks associated with boundary processing, which occurred sequentially in time (see Fig. 4, *SI Appendix*, Table S2, and Movie S1 for a visualization of the network interplay during boundary transitions). One network (IC_t=−1_) was significantly prominent immediately preceding the transition, while the other network (IC_t=0,1_) was active during the transition itself and continued into the postboundary period. The IC_t=−1_ network was a small-scale network, consisting primarily of bilateral posterior auditory cortical areas (PTC) with a right-hemispheric emphasis. For simplicity, we will refer to this network as the “Early Auditory Network.” In contrast, the IC_t=0,1_ network, which was engaged during and immediately after the boundary transition, was comparatively a larger-scale network, composed of more middle and anterior auditory areas and Rolandic opercula, both bilaterally. Concurrently, these activations were coupled with the deactivation of prefrontal areas including VLPFC (rightward bias in spatial extent), bilateral OFC, and DLPFC (with a leftward bias), along with bilateral PTC, PPC, MOG, and left cerebellum. For simplicity, we will refer to this network as the “Boundary Transition Network” as it is specifically engaged during and immediately after the boundary transition (Fig. 5 illustrates the significance of these networks as they relate to the shift in boundary processing; refer to *SI Appendix*, Table S2 for a comprehensive list of brain areas involved in these networks).

### Causal Influences from the Early Auditory to the Boundary Transition Network.

In line with Sridharan et al. (8), we aimed to test the hypothesis that the distinct networks underlying different phases of event boundary processing may exert directional or causal influence. Specifically, we predicted that the Early Auditory Network, which was active prior to the boundary transition, would exert directed or causal influence on regions within the Boundary Transition Network, associated with processing the boundary from transition onward. To this end, and in the context of functional connectivity analyses, GCA can help us elucidate the temporal ordering of brain activity, which can provide valuable insights into the underlying neural mechanisms of boundary processing. This involves identifying whether one brain region or network is driving the activity of another.

If $X_{n}$ and $Y_{n}$ are IC time courses for the two networks of interest (Early Auditory and Boundary Transition networks), Granger causality can determine the predictive value of unique information in one series to forecast the other’s values. If past values of X help forecast Y’s current value, we say that X Granger-causes Y.

GCA was performed on the backreconstructed subject-level ICA temporal courses associated with each functional network separately for each subject. The optimal model for GCA was estimated using akaike information criterion (AIC). In the context of fMRI signals, GCA faces a bidirectionality problem where unidirectional influence can turn into bidirectional interaction due to low temporal resolution and hemodynamic blurring (19). This can lead to both signals mutually Granger-causing one another and to an inflation of the measure of influence between the two. To address this issue, a difference of influence term ($F_{x\to y}-F_{y\to x}$) was estimated for the network pair under examination for every participant [(20); see Granger causality analyses for details on significance estimation].

The difference in the strength and direction of causal influence between the two networks indicated a unidirectional causal influence (two-sample Wilcoxon signed rank test, alpha = 0.0001, FDR-adjusted), where the Early Auditory Network Granger-caused the Boundary Transition Network across all participants.

### Musicians and Nonmusicians: Differences in Boundary Processing.

**Fig. 6.**
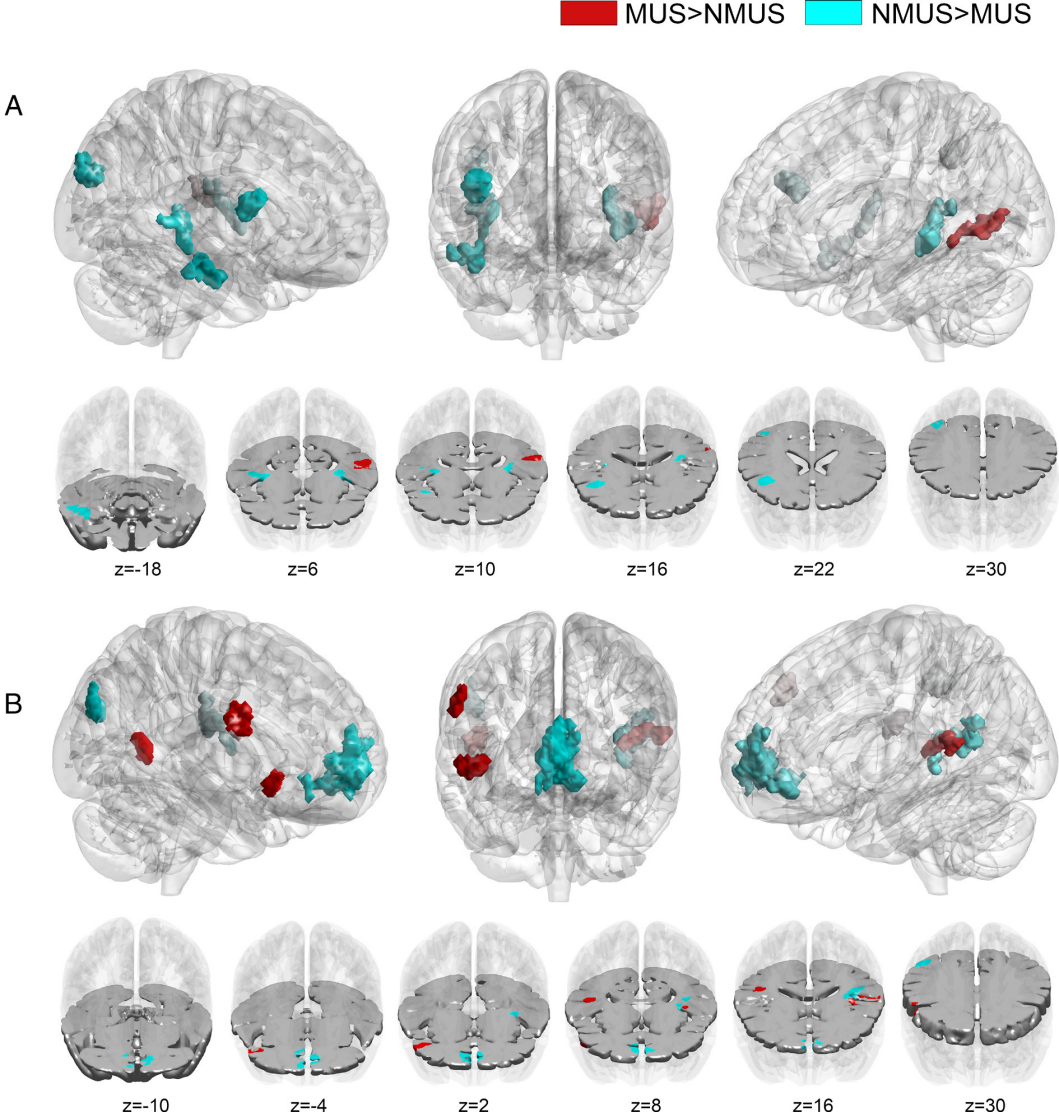
Musicians and nonmusicians: differences in boundary processing. Group differences in boundary processing during the moments leading to the transition (*A*; Early Auditory Network) and during and beyond the transition (*B*; Boundary Transition Network) revealed by ICA analyses (two-sample Wilcoxon signed rank tests, *P* < 0.05, cluster-wise corrected, FWE = 0.05).

The results of GLM analyses comparing regional activation between musicians and nonmusicians yielded no significant differences at any of the boundary shifts (t = −1, t = 0, and t = +1 lags; lag = scan interval = 2 s; two-tailed, two-sample Wilcoxon rank sum tests, alpha = 0.05; see Fig. 6 and *SI Appendix*, Table S3). The GLM approach thus does not indicate any significant influence of musicianship on regional hemodynamic responses during boundary transitions.

However, the results obtained through ICA tell a more nuanced story. By examining both the early auditory and Boundary Transition Networks, we identified statistically significant differences in network integration, i.e., degree of functional connectivity between brain regions, between musicians and nonmusicians.

Before boundary transitions, musicians exhibited increased network integration within the left MTG (BA22) while nonmusicians showed increased integration within a wider network including the bilateral Heschl’s gyrus, the right homologue of Broca’s area (BA44/45) and the right middle occipital gyrus (MOG, BA39). In contrast, at boundary transitions and onward, musicians exhibited more connectivity in the auditory cortices bilaterally (primary auditory & surrounding cortex) and right Broca’s area’s homologue (BA44/45), whereas nonmusicians showed increased integration within the left auditory cortex, the right medial prefrontal cortex (mPFC; BA10), and the right angular gyrus (BA39). Refer to *SI Appendix*, Table S3 for a comprehensive list of brain areas involved in these networks.

Overall, these results suggest distinct boundary processing strategies employed by musicians and nonmusicians, as reflected in their differential patterns of brain connectivity during music listening.

The ICA results align with additional analyses we conducted, as suggested by a reviewer, prompted by the absence of significant differences across lags or between groups in the GLM analysis. We further explored the GLM maps using agglomerative hierarchical clustering analyses, which revealed a clustering pattern consistent with the ICA results. Both musicians and nonmusicians exhibited clustering of lag 0 and lag +1 maps together, separate from lag −1. For detailed findings, please refer to *SI Appendix*, *Supplementary Methods*.

## Discussion

### General Findings and Comparison with Sridharan et al. (8).

While building upon Sridharan et al.’s (8) groundwork, our study focused on exploring boundary transitions at a finer level, specifically at the musical phrasal level. In contrast, Sridharan and colleagues primarily investigated boundary transitions at a coarser level, such as symphonic movement transitions, revealing two distinct brain networks—an early ventral network for event detection and a later dorsal network for information processing and working memory maintenance. These networks were causally linked from ventral to dorsal regions.

The present study aimed to capture a distinct phenomenon in boundary processing, considering the complexities of finer-grained boundaries. Similar to Sridharan et al. (8), we observed two statistically significant functional networks during the boundary transition, potentially indicating the involvement of distinct cognitive processes. The first network showed prominence before the transition, while the second network engaged during the boundary and extended into the postboundary period. Sequential activation and a causal link between the networks were revealed through Granger causality analyses.

Unlike Sridharan et al. (8), our study did not find a clear ventral to dorsal shift in recruited areas. This discrepancy may be attributed to the finer-grained transitions studied in our research, which might not align with the broader symphonic movement transitions examined by Sridharan et al. (8). Additionally, our study aimed to encompass three distinct styles within the Western musical tradition, in contrast to the specific focus on classical symphonies in their research. Consequently, our results may have broader generalizability and be extended to different musical styles. Nevertheless, it is important to exercise caution when extending our findings to other musical styles beyond this scope.

Our study identified an Early Auditory Network, primarily involving posterior auditory areas bilaterally (PTC), that became maximally activated prior to boundary transitions. In contrast, the Boundary Transition Network, active during and after the transition, encompassed middle and anterior auditory areas, Rolandic opercula, and showed deactivation of several regions, including notable prefrontal areas. Moreover, our results revealed a shift in auditory processing from posterior to middle and anterior areas during the transition from closing one phrasal boundary to processing a new boundary. This shift mirrors the posterior-to-anterior information flow observed during auditory sentence comprehension, where information flow starting in the posterior auditory cortex proceeds to the anterior STG, ultimately connecting to the frontal cortex through ventral pathways (21), and suggests a comparable process at work in our study. Additionally, this shift coincides with a coupled deactivation in frontal regions, consistent with previous language processing studies and our Granger causality analyses.

One intriguing finding was the deactivation observed in the right homologue of Broca’s area. While Broca’s area is a well-recognized neural substrate for speech production and grammar acquisition (22–24), its mirror counterpart in the right inferior frontal gyrus operculum (BA44/45) appears to engage in various nonlinguistic processes, such as musical syntax processing (25–28) and nonverbal auditory working memory (29–32).

The deactivation of the right fronto-opercular area during the transition and subsequent phases of boundary processing is noteworthy, considering its established involvement in music-related syntax and working memory. This deactivation aligns with the time when one would expect attention and working memory for musical events to be engaged. One interpretation is that the deactivation of the right VLPFC reflects a redirection of attention away from internal cognitive processes or a shift in focus toward external stimuli. Engaging in attention-demanding tasks often involves regional deactivations, indicating a decrease in neural activity within regions that support processes unrelated or irrelevant to the current task (33, 34). This observation is consistent with a recent review of the DMN (35), which emphasizes the significant role of DMN suppression in facilitating adaptive disengagement during tasks that demand external focus. These findings, at a minimum, imply an indirect contribution to cognitive processes through intricate interactions with other brain networks, underscoring the dynamic nature of brain networks and their roles in regulating attention and cognitive processes across various tasks.

Expanding on this interpretation, the VLPFC’s activation might be expected to vary depending on the listener’s level of analysis or immersion in the music. For instance, increased activation may occur during active analysis, involving cognitive processes such as evaluative judgments. However, during immersive listening experiences that emphasize aesthetics and emotions, the VLPFC may show reduced activation or deactivation, potentially reflecting a shift of attention away from internal cognitive processes to allow listeners to fully engage with the music. While the VLPFC’s direct association with emotion-processing regions such as the limbic cortex may be limited, its deactivation during immersive music listening experiences could still exert an indirect influence on emotional processes. For instance, in our study, the deactivation of the VLFPC was concurrently coupled with the bilateral OFC, and area extensively connected with various neural regions crucial for regulating motivations, emotional and social behaviors (e.g., including medial temporal cortical areas, hypothalamic and brainstem autonomic areas, and the amygdala) (36, 37). We acknowledge that these inferences are speculative given the lack of emotional ratings from participants, therefore, further empirical exploration is necessary to solidify their validity and delineate their implications.

A significant aspect to mention is the right lateralization of prefrontal areas observed in our study. This is consistent with the right hemispheric dominance for pragmatic aspects of language, such as interpreting prosodic features like accentuation and boundary markings expressed through pitch variations (38–41). This alignment with previous research supports the notion that music and language share common neural mechanisms in processing these aspects of auditory communication (27, 42).

Last, the involvement of the Rolandic operculum in this context deserves special attention, given its association with sensorimotor functions, crucial for planning and executing voluntary movements (43). Within the Boundary Transition Network, the Rolandic operculum exhibited bilateral coactivation with the auditory cortical areas. Moreover, the Rolandic operculum encompasses specific subregions known to play a crucial role in the control and coordination of speech and vocalization-related movements. These include the representation of articulatory muscles involved in speech production (31). Notably, the Rolandic operculum includes the ventral part of the larynx motor cortex in the left hemisphere (44). Hence, it is plausible that the activated portion of the Rolandic operculum in the present study may potentially contain the representation of the larynx (z = 8 to 14; MNI coordinates). This observation bears relevance as it hints at a possible association between the activated portion of the Rolandic operculum in our study and larynx-related processes, potentially indicating a form of perception-execution matching (31). This may indicate motor involvement in processing musical stimuli, resembling internal generation of melody and rhythm, akin to covert (inner) singing. Such activation is likely to occur at the onset phase of boundary transitions as listeners generate predictions and anticipate musical events. More recent fMRI evidence shows that both speaking and hearing vowel sounds activate sensorimotor areas activating a left brain pathway that connects auditory perception with speech articulation, including the rolandic operculum (45). This directly implicates the Rolandic operculum in processing both the production and perception of vocalizations, hinting at a potential laryngeal representation within this region.

In light of these findings, the engagement of the Rolandic operculum during and after boundary transitions may suggest its involvement in facilitating preparatory signals potentially linked to sensorimotor processes such as action imitation and rhythmic entrainment. However, it is essential to acknowledge that this interpretation remains speculative at this stage. Future research could explore this hypothesis further and implement measures to control for potential confounding factors, such as subvocalizations, to provide a more conclusive understanding of the observed activations.

In summary, our findings highlight two different networks at different stages of the boundary transition, potentially reflecting distinct cognitive processes. Before a boundary transition, an anticipatory network prepares for upcoming changes. During and after the transition, a different network integrates the new information while updating the mental representation of the musical phrase. These networks support the processing and updating of the musical representation, ensuring a coherent understanding of the musical structure.

### DMN Deactivation during Boundary Transitions.

In the context of musical phrase comprehension, the observed anticorrelated relationship between the auditory areas and the prefrontal cortex may potentially be related to the default mode network (DMN). The DMN is a network typically associated with introspection, mind-wandering, and internal mental processes (46). It tends to deactivate during external tasks or focused attention. This deactivation allows cognitive resources to shift toward externally focused and goal-directed processes.

This significant shutdown of regions associated with DMN areas seems to underscore a concurrent interaction with auditory processing areas in the present study. Such polarity between auditory and the DMN could suggest an anticorrelated or excitatory–inhibitory relationship, where the activation of one region suppresses the activity of the other, indicating an interplay between auditory processing and introspective cognition. This would allow the brain to allocate resources more efficiently for processing musical boundaries and integrating incoming musical information. By potentially reducing introspective mental processes, the brain may enhance its ability to analyze structural changes at the music boundaries, thereby improving the effectiveness of music boundary processing.

A possible explanation for this phenomenon is that a trade-off occurs between sensory processing and cognitive control. Essentially, when we are anticipating and paying close attention to what we are listening in the music, the brain may prioritize sensory areas over higher-order cognitive regions to efficiently process incoming information. This prioritization may potentially place a demand on sensory processing, such as the auditory areas, resulting in a temporary disengagement of higher-order cognitive areas. This interpretation emphasizes the dynamic interplay between sensory and cognitive systems during music perception and recognizes the necessary trade-off between efficient sensory information processing and cognitive control.

Adding to this, the anticorrelated relationship between the auditory areas and the DMN areas is also supported by Granger causality analyses. The information flow from auditory areas to the prefrontal cortex suggests that auditory engagement may inhibit the DMN during boundary transitions via inhibitory signals. Taken together, further investigation would be necessary to fully understand the implications of the anticorrelated relationship in the context of the DMN.

### Effect of Musicianship.

The present analyses indicated differences in the engagement of brain networks between musicians and nonmusicians before and during/after boundary transitions. These findings align with consistent research results, which highlight the influence of musical training on the perception of music phrase structure (47–50).

Before boundaries, musicians showed increased network integration within the left MTG (BA22), whereas nonmusicians showed increased integration across a broader network encompassing auditory cortices, right Broca’s area homologue (BA44/45), and right middle occipital gyrus (MOG, BA39). During and after boundaries, the activation pattern shifted. Musicians exhibited enhanced network connectivity within the auditory cortices, left Rolandic operculum, as well as the right Broca’s area’s homologue (BA44/45), while nonmusicians displayed increased integration within the left auditory cortex, right mPFC (BA10), and right angular gyrus (BA39).

In the context of music listening, activations observed in the right fronto-opercular area (BA44/45) are believed to contribute to music-syntactic analysis and the detection of harmonic rule violations, as discussed earlier. Considering the background research on the role of the right hemispheric counterpart of Broca’s area, the differential engagement observed in nonmusicians and musicians during different transition phases suggests distinct boundary processing strategies. Nonmusicians relied more on this region more prior to boundary transitions as part of a broader network, potentially involving cognitive control, to prepare for musical changes indicated by a boundary transition. Conversely, musicians showed increased engagement of the right Broca’s area’s homologue alongside other auditory processing and motor control regions during and after boundary transitions. This enhanced engagement in musicians can be attributed to their music expertise and the integration of auditory and motor processes essential for playing instruments or performing musical tasks. This, in turn, may aid in predictive listening at the onset of a musical phrase and beyond.

Adding to this, a study by Neuhaus et al. (49) identified processing differences in boundary processing between musicians and nonmusicians using event-related brain potentials (ERPs) and event-related magnetic fields (ERFs). Musicians showed distinct ERPs and ERFs compared to nonmusicians when listening to phrased melodies suggesting a structured processing similar to language, while nonmusicians focused more on detecting melodic discontinuity. Moreover, a study conducted by Saari et al. (51), using a decoding approach, found that the right Broca’s homologue plays a crucial role in achieving optimal classification accuracy for listeners’ musicianship class based on their fMRI responses to music. The authors stressed the sensitivity of the right Broca’s homologue to musical training and its impact on the processing and detection of regularities in music.

Overall, the right Broca’s area homologue appears to be involved in both groups, but its specific role may vary depending on the context and the individual’s musical expertise. In nonmusicians, it could be related to cognitive control, while in musicians, it may facilitate the integration of auditory and motor processes during musical listening via action simulation mechanisms. This is particularly evident as musicians develop associations between instrumental movements and their auditory effects (52).

With regard to the engagement of the Rolandic operculum observed in musicians, it could suggest action simulation processes such as covert singing or imagining instrument playing. Action simulation involves mentally simulating or simulating actions in response to sensory input. The Rolandic operculum, located in the frontal lobe, is associated with motor functions, including action planning and execution (43). Musicians, with their extensive training and experience, likely possess well-developed motor representations and cognitive processes related to actions. The enhanced connectivity within the auditory cortices and the left Rolandic operculum suggests that musicians may engage in mentally simulating musical actions, such as playing an instrument or vocalizing. This interpretation makes particular sense if we consider that the activated portion of the Rolandic operculum may contain representations of the larynx, further supporting the notion of action simulation during music listening. Supporting this, covert singing has been associated with activity around the larynx area (53). Similarly, investigations into musical discrimination, which often lack explicit subvocalization components, have demonstrated activation of the larynx area during tasks such as same/different discriminations of pairs of melodies (54).

In sum, results could be explained by the impact of differences in expertise on cognitive strategies. Musicians, being attuned to the musical cues and patterns present during the transition, exhibit greater engagement of the right Broca area and action simulation mechanisms (left Rolandic operculum). Nonmusicians, on the other hand, exhibit a broader, more generalized response, relying on general cognitive processes to navigate the uncertainties and ambiguities during the transition. Their engagement of the PFC, auditory cortex, and parietal cortex to a greater extent than musicians may reflect the use of these general cognitive processes to face the demands of predicting the musical flow (55). Taken together, these findings highlight the importance of considering individual musical expertise when studying cognitive processes related to boundary transitions.

### Predictive Uncertainty, Boundary Processing, and Musicianship.

Consensus in the literature emphasizes the role of prediction error or surprise in event parsing (56–60). Behavioral studies support this, showing that individuals are capable of hierarchically segmenting events and adjusting their segmentation processes based on the uncertainty surrounding ongoing events (1, 61, 62). Previous research in information dynamics highlights that boundaries in music and speech are perceived at points of unpredictability or unexpectedness, aligning with expectations (63–65).

**Fig. 7.**
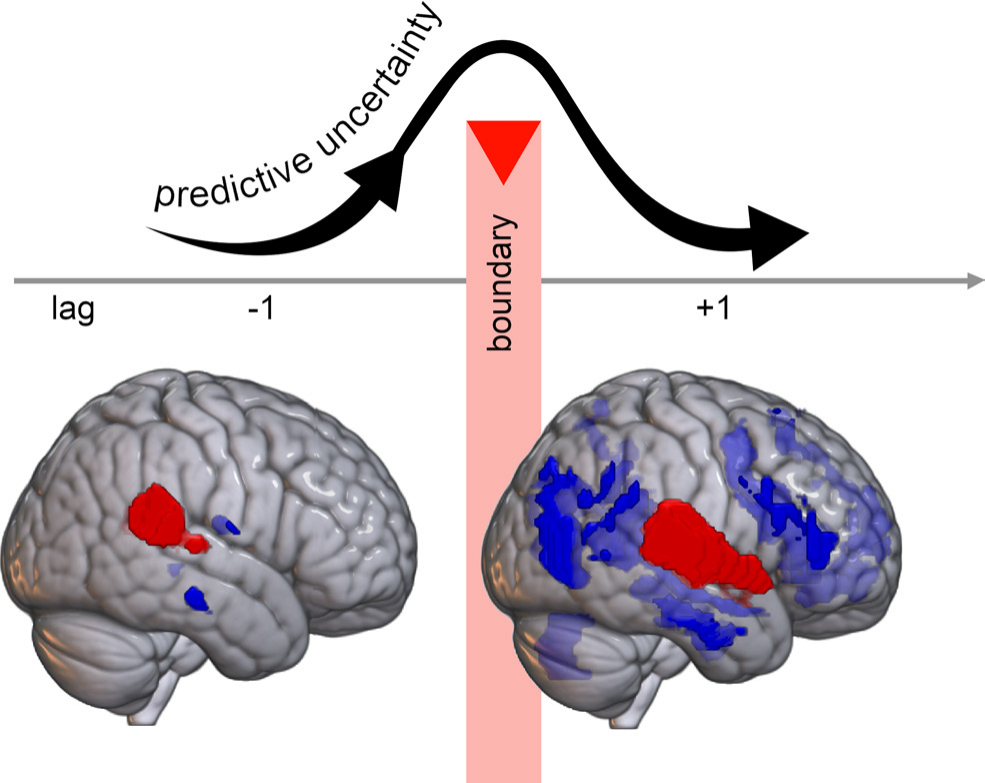
Predictive uncertainty fluctuates during boundary phases, influencing the interplay between sensory and cognitive systems. As the boundary approaches, uncertainty intensifies, as there is limited information available to predict forthcoming events, hence boundaries can be construed as peaks of uncertainty. Subsequently, immediately after the boundary, uncertainty gradually decreases, prompting the brain to generate predictions and adjust them accordingly. This reflects the brain’s adaptation to the unfolding musical events.

Building on this understanding, the relationship between sensory and cognitive areas during boundary transitions can be understood within the context of prediction uncertainty. Predictive uncertainty refers to an individual’s subjective state of expectation regarding potential outcomes before an event occurs, influenced by the interaction between anticipation and internal cognitive models (55). These models generate conditional probability distributions based on preceding events and refine through experience, with accurate models leading to stronger predictions. Accurate models lead to stronger predictions, while less accurate models struggle in providing certainty, especially in complex or unfamiliar situations (i.e., high-entropy contexts). However, approaching a boundary disrupts the ongoing pattern, reducing music predictability and increasing predictive uncertainty. In response, the brain must adapt and adjust its predictions to accommodate this shift (Fig. 7).

The immediate moment preceding the boundaries can be construed as related to musical resolution or even silence, particularly in coarse-grained transitions, as in Sridharan et al. (8). As a consequence, there is limited information available to predict forthcoming events (66), potentially requiring less action simulation. This phenomenon is evident at the onset of the boundary (Boundary Transition Network) with the involvement of the Rolandic opercular areas.

In a state of gradually decreasing uncertainty following the onset of a new boundary, the listener is afforded the opportunity to explore and entertain various predictions regarding the unfolding of the music. This adjustment may involve a shift in attention from cognitive control to sensory processing, enabling the brain to process and integrate the new information more effectively. The polarity between sensory and cognitive areas, characterized by an inhibitory relationship, facilitates this process, optimizing processing and minimizing prediction errors in the face of uncertainty. This interaction is crucial not only for perceiving and comprehending music but also for understanding other complex stimuli, emphasizing the broader significance of how the brain integrates information across various processing levels.

The musicianship factor can also be examined in the context of predictive uncertainty. Musicians, through years of experience in music practice and theory, tend to develop enhanced representations of musical regularities (musical grammar) and have high sensitivity to deviations from these regularities (67). This expertise allows musicians to make robust predictions about forthcoming musical events, particularly in low-entropy or high predictable contexts (68). As a result, their cognitive models are optimized to navigate the intricate complexities and nuances of music, leading to increased accuracy and reduced predictive uncertainty compared to nonexperts.

However, this optimized cognitive processing comes with a trade-off. When confronted with unexpected auditory events, musicians’ finely tuned ability to quickly detect and process discrepancies between predictions and auditory input results in heightened neural responses to prediction errors (69–75). This phenomenon has not been observed to the same extent in nonmusicians (70, 72, 74–77). Such observation provides a plausible explanation for the differential processing of boundaries between musicians and nonmusicians particularly within the right fronto-opercular areas.

Moreover, it is worth highlighting that previous studies consistently link the VLPFC to detecting violations in musical expectancies, even in nonmusicians (25, 78), suggesting the ventral network’s role in detecting differences between expectations and sensory events (79, 80). These discrepancies are critical for event segmentation, as boundaries are perceived when transient prediction errors occur (81). Hence, the observed group differences may reflect musicians’ predictive processing advantages and the strategies employed by musicians as experts in the music domain and its syntax.

## Conclusions

The ability to perceive boundaries is not only vital for our understanding of music and art but also essential for our most basic everyday functioning. As we listen to music, our brains process the structure by identifying patterns and predicting what will happen next. Our perception of phrasal boundaries becomes then critical for understanding the structure and meaning of musical compositions. The present study investigated listeners’ spatiotemporal brain dynamics in listeners during event segmentation of fine-scale musical boundaries and studied the impact of musical training.

Our study addresses limitations in understanding boundary processing by adopting a paradigm that investigates this phenomenon without interrupting the stimulus or neglecting the complexities or realistic environments, providing valuable insights into this important aspect of auditory perception. Additionally, our flexible methodological approach in ICA analysis allows for a comprehensive exploration of neural processes without constraining the analysis to a specific, predetermined model order. This dynamic determination of the most suitable model order enhances the reliability and validity of our findings.

The findings demonstrate the neuroplasticity of the human brain and how expertise can shape and refine our neural processing. The data suggest the involvement of specific brain mechanisms that listeners employ to process continuous music at the phrasal level. Despite the observed polarity between sensory auditory areas and prefrontal cortical areas during boundary transitions, the exact mechanisms and functional significance of this relationship remain unresolved. The question of how sensory and cognitive areas interact and integrate during boundary transitions is a relevant one. Further research is needed to understand the intricate interplay between these brain regions and their role in music perception and cognition.

One key observation is that, much like the processing of language, the processing of musical phrases activates the auditory cortex and other associated regions involved in syntactic processing and rule detection, underscoring the existence of a language-like system for music in the brain. This parallel emphasizes the intricate and interconnected nature of human cognition, where various forms of communication, including music, engage common neural substrates for processing and comprehension. The results also emphasize the intertwined nature of sensory and cognitive systems during music boundary processing and the importance of striking a balance between efficient processing of sensory information and cognitive control.

Future investigations could include control measurements at moments not perceived as boundaries, but containing non-boundary features. For instance, observing brain activity during attention-grabbing events not signaling a boundary, such as a bell, or during moments of silence perceived as expressive rhythmic elements rather than boundaries, could provide insight into the exclusivity of neural markers associated with musical boundaries versus other elements in the music.

This research lays the groundwork for further investigations into the functional connectivity, perceptual correlates, and cognitive implications of boundary processing across diverse populations and musical contexts. By delving deeper into these areas, we can gain valuable insights into the intricate interplay between musical structure, expertise, and cognitive processing, ultimately advancing our knowledge of how the brain engages with and makes sense of music.

## Materials and Methods

A detailed description of methods used in this study is available in *SI Appendix*, *Supplementary Methods*. In brief, GLM analyses were performed by shifting the boundary regressor in time by one scan (2 s) before and after the boundary location, and GLM results were pooled across all participants using Fisher’s combined probability test to examine the common brain pattern of activation for both musicians and nonmusicians. Between-group comparisons were assessed by means of two-sample Wilcoxon signed rank tests. Spearman’s rank correlation was used as an alternative method for assessing the association between the boundary regressor and fMRI responses to avoid making any assumptions about specific distributions or linearity between variables. Spatial ICA analyses were performed within the selected ROI using a two-step dimensionality reduction procedure (both at the individual and group level), following a decomposition that did not rely on prior assumptions about the model order (i.e., the latent dimensionality of the data). Subject-level statistical inference was enabled by applying the GICA3 algorithm, which allows the reconstruction of subject-specific IC spatial maps and temporal courses. The optimal model order for Granger causality analyses was determined using both the AIC and the Bayesian Information Criterion (BIC). A difference of influence term was used to address spurious causal influences in fMRI signals caused by low temporal resolution and hemodynamic blurring. The experiment was undertaken with the understanding and written consent of all participants. The study protocol proceeded upon acceptance by the ethics committee of the Coordinating Board of the Helsinki and Uusimaa Hospital District.

## Supplementary Material

Appendix 01 (PDF)

Movie S1. **Dynamic brain changes during boundary transitions.** Movie S1 exhibits the engagement of the Early Auditory Network and Boundary Transition Network, highlighting the dynamic shifts in functional connectivity and adaptive responses of the two networks during the processing of musical boundaries (t = -1, 0, and +1, representing moments before, during, and after the boundary transition (t = TR = 2s). The smooth transition between these two distinct functional networks was achieved through the utilization of 3D linear interpolation for visualizing purposes. Three musical excerpts representing boundary transition peaks were selected per musical stimuli to highlight the dynamic shifts in functional brain networks.

## Data Availability

Due to ethical regulations governing the data collection based on the protocol approved by the Coordinating Committee of the Helsinki and Uusimaa Hospital District, the original fMRI dataset used in the present study can be accessed by qualified investigators only upon individual research data sharing agreements. Please coordinate with the main author (iballa.burunat@jyu.fi). Other data supporting the paper’s findings can be found in the article and its supporting information. Select data and code are archived in Zenodo (https://doi.org/10.5281/zenodo.10865354) (82).
