# Supplementary material for: Breaking (musical) boundaries by investigating brain dynamics of event segmentation during real-life music-listening
Source: Proc Natl Acad Sci U S A. 2024 Aug 26;121(36):e2319459121. doi: 10.1073/pnas.2319459121 (PMC11388323; doi:10.1073/pnas.2319459121)
Supplement: Supplementary file 1 — Appendix 01 (PDF) [file pnas.2319459121.sapp.pdf]

PNAS

Supporting Information for

Breaking (Musical) Boundaries By Investigating Brain Dynamics Of Event Segmentation During Real-Life Music-Listening

Iballa Burunat[a], Daniel Levitin[b,c], & Petri Toiviainen[a]

Iballa Burunat

Email: iballa.burunat@jyu.fi

This PDF file includes:

Supplementary Methods
Statistical Analyses
Abbreviations used in the Tables
Figures S1 and S2
Tables S1 to S3
Legends for Movie S1
SI References

Other supporting materials for this manuscript include the following:

Movie S1

Supplementary Methods

Participants

FMRI experiment

Thirty-six healthy participants with no history of neurological or psychological disorders participated in the fMRI experiment. The participants were screened for inclusion criteria before admission to the experiment (no ferromagnetic material in their body; no tattoo or recent permanent coloring; no pregnancy or breastfeeding; no chronic pharmacological medication; no claustrophobia). The participant pool was selected to include an equal number of professional musicians (n = 18, age = 28.2±7.8, females = 9) and nonmusicians (n = 18, age = 29.2±10.7, females = 10, left handers = 1). The criteria for musicianship were having more than 5 years of music training, having finished a music degree in a music academy, reporting themselves as musicians, and working professionally as a performer. As for the type of musicians, there were classical (n = 12), jazz (n = 4), and pop (n = 2) musicians. The instruments played were strings (violin = 4; cello = 2; double bass = 1), piano (n = 8), winds (trombone = 1; bassoon = 1), and mixed (n = 1). The musicians' group was homogeneous in terms of the duration of their musical training, onset age of instrument practice, and amount of years of active instrument playing. These details were obtained and crosschecked via questionnaires and Helsinki Inventory for Music and Affect Behavior (HIMAB; Gold et al., 2013). All participants classified as musicians had to meet all four criteria without exception, while nonmusicians were disqualified from meeting any of the four criteria. Both groups were comparable with respect to gender, age distribution, cognitive performance, socioeconomic status, and personality and mood questionnaire. The present dataset was part of a broad project ("Tunteet") investigating different hypotheses related to auditory processing and its dependence on person related factors by means of a multidimensional set of paradigms and tests, involving several experimental sessions, brain and behavioral measures as well as questionnaires. The findings related to the various hypotheses investigated appear in separate papers (cf. 2-4).

Perceptual experiment

Thirty-six participants took part in the perceptual experiment (18 nonmusicians [females = 8] and 18 musicians [females = 10]). The mean age of the participants was 27.45 years (SD = 4.54). They were all students or graduates from different faculties of the University of Jyväskylä and of the JAMK University of Applied Sciences. Participants were rewarded with a movie ticket as a token for their participation. Both musicians and nonmusicians met the relevant group criteria outlined previously. Musicians had an average of 14.39 years (SD = 7.49) of musical training. The musical style played by 12 of the musicians was classical music, whereas the other 6 musicians played non-classical musical styles. The main instruments played by participants were piano (5), guitar (4), flute (2), bass guitar, clarinet, saxophone, cello, violin, viola and voice. All the nonmusicians reported having had no musical training, whereas all of the selected musicians considered themselves either as semiprofessional (12) or professional (6 participants) musicians at the time of the data collection. None of the participants reported experience in dance, ballet or sound engineering. Six participants were very familiar with at least one stimulus but nobody reported having performed any of the examples. As a general rule, we referred to a participant as musician when they had reported more than 8 years of musical training and had also considered themselves as semiprofessional musician or professional musician. We discarded, for example, participants who, in a multiple-choice questionnaire, reported to be amateur musicians. In contrast, we considered participants to be nonmusicians if they considered themselves as nonmusicians and if they did not report any musical training.

Stimuli

Three musical pieces were used in the experiment: (a) Stream of Consciousness by Dream Theater; (b) Adios Nonino by Astor Piazzolla; and (c) Rite of Spring (comprising the first three

episodes from Part I: Introduction, Augurs of Spring, and Ritual of Abduction) by Igor Stravinsky. These are a progressive rock/metal piece, an Argentinian New Tango, and an iconic 20th century classical work, respectively, thus covering distinct musical genres and styles. All three selected pieces are instrumental and have a duration of about 8 minutes:

1. Piazzolla Piazzolla, A. (1959). Adiós Nonino. Recorded by Astor Piazzolla y su Sexteto. On The Lausanne Concert [CD]. BMG Music (1993). Spotify link: http://open.spotify.com/track/6X5SzbloyesrQQb3Ht4Ojx (excerpt: 0-08:07.968).
2. Dream Theater Petrucci, J., Myung, J., Rudess, J. & Portnoy, M. (2003). Stream of Consciousness (instrumental). Recorded by Dream Theater. On Train of Thought [CD]. Elektra Records. (2003) Spotify link: http://open.spotify.com/track/3TG1GHK82boR3aUDEpZA5f (excerpt: 0-07:50.979).
3. Stravinsky, I. (1947). The Rite of Spring (revised version for Orchestra) Part I: The Adoration of The Earth (Introduction, The Augurs of Spring: Dances of the Young Girls, Ritual of Abduction). Recorded by Orchestra of the Kirov Opera, St. Petersburg, Valery Gergiev. On Stravinsky: The Rite of Spring / Scriabin: The Poem of Ecstasy [CD]. Philips (2001) Spotify link: http://open.spotify.com/album/22LYJ9orjaJOPi8xl4ZQSq (first three tracks; excerpt: 00-07:47.243).

FMRI scanning and preprocessing

Participants' brain responses were acquired while they listened to each of the musical stimuli in a counterbalanced order. For each participant the stimuli loudness was adjusted to a comfortable but audible level inside the scanner room (around 75 dB). In the scanner, participants' only task was to attentively listen to the music delivered via high-quality MR compatible insert earphones while keeping their eyes open.

Scanning was performed using a 3T MAGNETOM Skyra whole-body scanner (Siemens Healthcare, Erlangen, Germany) and a standard 20-channel head-neck coil, at the Advanced Magnetic Imaging (AMI) Centre (Aalto University, Espoo, Finland). Using a single shot gradient echo planar imaging (EPI) sequence, 33 oblique slices (field of view = 192x192 mm; 64x64 matrix; slice thickness = 4 mm, interslice skip = 0 mm; echo time = 32 ms; flip angle = 75°) were acquired every two seconds, providing whole-brain coverage. T1-weighted structural images (176 slices; field of view = 256x256 mm; matrix = 256×256; slice thickness = 1 mm; interslice skip = 0 mm; pulse sequence = MPRAGE) were also collected for individual coregistration. Functional MRI scans were preprocessed on a Matlab platform using SPM8 (Statistical Parametric Mapping), VBM5 for SPM (Voxel Based Morphometry; Ashburner & Friston, 2000; Wellcome Department of Imaging Neuroscience, London, UK), and customized scripts developed by the present authors. For each participant, low resolution images were realigned on six dimensions using rigid body transformations (translation and rotation corrections did not exceed 2 mm and 2° respectively), segmented into grey matter, white matter, and cerebrospinal fluid, and registered to the corresponding segmented high resolution T1 weighted structural images. These were in turn normalized to the MNI (Montreal Neurological Institute) (6) segmented standard a priori tissue templates using a 12-parameter affine transformation. Spatial smoothing was then applied to the functional images using an 8 mm full width at half maximum Gaussian filter as to enhance the signal-to-noise ratio. Movement related variance components in fMRI time series resulting from residual motion artifacts, assessed by the six parameters of the rigid body transformation in the realignment stage, were regressed out from each voxel time series. Next, spline interpolation was used to detrend the fMRI data, followed by temporal filtering (Gaussian smoothing with kernel width = 4 sec).

Brain responses to the three stimuli were concatenated making a total of ~24 minutes worth of data. The rationale behind this was to combine stimuli representing a wide range of musical genres and styles in order to cancel out effects that the specific kinds of music may have on the phenomenon under investigation. The final time series had 702 samples after the 4 first samples of each of the three runs were removed to avoid artifacts due to magnetization effects.

Perceptual experiment to obtain boundary data

To determine the precise locations of the boundaries in the music (i.e., transition points), a perceptual experiment was carried out using a computer in a sound-attenuated room.

This perceptual task was conducted with a separate participant pool (see Perceptual experiment). The rationale for using a different participant pool for fMRI and perceptual experiments is that it allows to minimize familiarity effects with the music, which could affect the listening task during the fMRI scanning (or vice versa), leading to participants reacting differently to cadential closure, repetition, and other features that could contribute to boundary detection. However, to minimize differences, groups were matched in terms of their demographic variables.

Participants were instructed to mark 'instants of significant change' as they listened to the music by pressing the space bar of the computer keyboard in real time via a Max/MSP computer patch (7). During the experiment, the boundary data for each participant and stimulus was collected in a single pass, without any opportunity for them to preview the stimuli prior to segmentation, nor to make any changes to their boundary indications after completing the task. Subsequently, they were free to playback from different parts of the stimulus and make their segmentations more precise by adjusting the position of boundaries or remove them if these were added by mistake. However, participants could not add any new boundaries at this stage.

The interface included a play bar that offered basic visual-spatial cues regarding the beginning, current time position and end of the examples.

To avoid effects of fatigue, each stimulus was presented to participants as four musical extracts:

- Piazzolla: 0-02:00, 01:57-03.57, 03:54-05:54, 05:51-08:07.968.
- Dream Theater: 0-02:00, 01:57-03.57, 03:54-05:54, 05:51-07:50.979
- Stravinsky: 00:05-02:05, 02:02-04:02, 03:59-05:59, 05:56-07:52.243

Subsequently, participants' segmentation responses were combined to obtain a set of indicated boundaries for the complete stimulus. Kernel Density Estimation (KDE) (8) was applied to the vector of aggregated boundary indications for each participant group. KDE involves centering a Gaussian kernel at each boundary and summing the kernels to create a smoothed density curve. The resulting smoothed density curve reveals peaks where multiple listeners marked a boundary. Separate KDE computations were performed for musicians and nonmusicians to estimate the probability density curve. KDE achieves a smooth distribution by applying a kernel function (typically Gaussian) to each data point. For this study, a Gaussian kernel width of 1.66 seconds was selected (9). Between-group consistency was high ($r = .9$, $p < 0.001$). The KDE was computed with a sampling interval of 10 Hz.

The KDE time series for each group was convolved with the canonical double gamma hemodynamic response function (HRF) in order to match the hemodynamic response delay typical of blood oxygen level dependent (BOLD) brain responses, and downsampled to 0.5 Hz to match the sampling rate of the fMRI scanner. The peaks in this time series indicate the temporal locations where salient changes in the music materials occurred, as measured by high between-listeners consensus. We will refer to this variable as the “boundary regressor”.

Statistical analyses

Region of Interest (ROI) selection

To restrict the brain area to be examined, an initial GLM analysis was performed to identify brain regions significantly related to the boundary regressor (alpha = 0.001, cluster wise corrected, FWE = 0.05). The rationale for restricting the brain volume subjected to further analysis is twofold: (1) to reduce the issue of multiple comparisons by decreasing the number of statistical tests and only

including informative brain volume, and (2) to improve the separation and anatomical precision of the identified spatial components within the context of the ICA approach (10–12). Accordingly, the resulting ROI contained a total of 29980 voxels, and was used in the subsequent GLM and ICA analyses, to determine, respectively, both the region- and network-related brain activation driven by the processing of boundaries in the music.

GLM analyses

Following the approach used by Sridharan et al. (2007), we analyzed the moments before, during, and after the boundary transitions. That is, we examined regional activation related to the boundary regressor at different lags (0 lag ± 1 lags; each lag represents 2 seconds). This allowed us to investigate how brain activity changed leading up to, during, and following the transitions, and how this activity was temporally related to the onset of the boundary events.

To do this, we performed three GLM analyses on the selected ROI by shifting the boundary regressor in time by one scan (2 seconds) before and after the boundary location to identify regions predicted significantly by it ($p < 0.001$, cluster wise corrected, FWE = 0.05; see Figure 7 for an overall visualization of the statistical pipeline of the study).

GLM Analyses were performed separately for musicians and nonmusicians using the group-level boundary regressor specific to each group. The significance of the correlation coefficients was assessed by considering the intrinsic serial correlation between adjacent fMRI samples via estimation of the effective degrees of freedom (13). In line with this method, degrees of freedom were computed for each participant by randomly selecting 10,000 voxels iteratively and subsequently averaging the estimates across participants. GLM results were pooled across all participants (Fisher's combined probability test, $N = 36$, $p < 0.001$, cluster-wise corrected, FWE = 0.05) to examine the common brain pattern of activation for both musicians and nonmusicians during boundary transitions. Between-group comparisons to determine group differences in regional activation between musicians and nonmusicians were assessed by means of two-sample Wilcoxon signed rank tests (two-tailed, alpha = 0.05). Similar tests were performed to compare BOLD signal changes between the different shifts.

Nonparametric analyses

The normality assumptions may not always be fully met in fMRI data because the distribution of fMRI data is often complex and can be influenced by various factors (i.e., noise, artifacts, and the underlying neural activity being measured). It is important to note that the departure from normality does not necessarily invalidate the use of GLM in fMRI. Many statistical methods are robust to departures from normality, especially for large sample sizes. Additionally, the Central Limit Theorem (CLT) and the use of large sample sizes in fMRI studies can help mitigate the impact of departures from normality at the individual voxel level when pooling results across participants. This means that even if the individual fMRI data within each group may not be normally distributed, the distribution of group means or group-level statistics tends to approach a normal distribution as the sample size increases.

However, the GLM framework does assume a linear association between the predicted and predictor variables. If the relationship between variables is not linear, GLMs may not accurately capture the true association. Non-linear relationships can be assessed using alternative measures, such as rank-based correlation coefficients (e.g., Spearman's rank correlation) that do not assume linearity. Therefore, it was deemed appropriate to explore this a non-parametric technique in addition to GLM analyses.

We conducted an identical approach using Spearman's rank correlation analyses along with permutation tests for significance estimation. Spearman's rank correlation calculates the correlation between the ranks of the variables rather than their raw values, making it robust to non-linear relationships and outliers and providing a more flexible alternative to GLMs. This nonparametric approach provided an alternative method for assessing the association between the boundary

regressor and fMRI responses, without making any assumptions about specific distributions or linearity between variables.

To quantify the significance of the final Z-transformed Fisher's combined probability scores, a null distribution of z-scores under the null hypothesis of no fMRI vs regressor correlation was estimated using ‘random’ versions of the boundary regressor. Because merely randomizing the samples in the time series does not preserve its temporal dependencies, leading to biased estimates, a phase-scrambling procedure was employed (14). This involved scrambling the phases of the boundary regressor time series in the frequency domain and subsequently inverse-transforming them to the time domain. The significance of the z-scores was subsequently derived from this distribution.

The primary disparity identified in the spatial maps derived from the GLM and nonparametric methodology pertained only to the statistical significance. Specifically, the nonparametric analyses revealed a higher level of significance compared to the GLM method. For instance, voxels that attained a significance level of 0.001 (one-tailed) in the GLM approach exhibited a significance level of 0.0005 (one-tailed) in the nonparametric analyses. This was expected given the increased statistical power associated with the non-parametric test.

It is noteworthy however that the continuous spatial maps generated by both approaches showed a high correlation ($r > 0.96$, $p < 0.001$). These findings indicate a high degree of similarity in the spatial patterns of hemodynamic activity, with consistent spatial relationships observed between the maps. Consequently, both GLM and nonparametric maps exhibited identical clusters but expanded in size in the nonparametric approach. This discrepancy may be attributed to the enhanced sensitivity of the nonparametric approach in detecting subtle activation patterns, as well as its ability to relax the assumption of linearity imposed by the GLM.

Agglomeratice Hierarchical Clustering of GLM maps

Following one reviewer's suggestion, prompted by the absence of significant differences across lags or between groups in the GLM analysis, we delved deeper into the GLM results by employing aglglomerative hierarchical clustering analyses. This technique helped cluster the GLM maps, providing insights into their similarity. We used the GLM continuous z-score spatial maps corresponding to each lag in vectorized form, averaged across lags, and calculated the cosine distance between these vectors, generating a pairwise distance matrix. Subsequently, we applied the Ward linkage method to this matrix to construct a hierarchical clustering tree. The resulting dendrogram was then generated (see Figure S1). This method facilitated the elucidation of spatial relationships among lag-specific z-score maps, aiding in the interpretation of our findings.

Subsequently, we conducted an separate identical analysis using group averages per lag (musicians and nonmusician). Notably, both groups exhibited consistent clustering of lag 0 and lag +1 maps, distinct from lag-1, aligning with prior results (see Figure S2). These findings confirm earlier observations while also incorporating musician-nonmusician distinctions. We observed identical results when employing the Euclidean distance metric in conjunction with the average linkage method.

ICA analyses

Spatial ICA

Spatial ICA (sICA) (15) was applied to the ROI selection, following an identical pipeline as described in (23). This variety of ICA is commonly performed in fMRI, where it decomposes the fMRI signal into spatially independent components (ICs). The basic assumption in ICA is that each voxel time series (observation) represents a linear combination of a number of unknown underlying source signals, which are assumed to be maximally statistically independent from one another. In the case of sICA, the assumption of independence means that the identified IC spatial maps do not overlap in space (16). Each IC may thus represent a functionally coherent brain network (e.g., visual, auditory, or motor), a physiological process (e.g., breathing) or an artefact (e.g., head motion) (17).

Prior to performing ICA, participants' data were reduced both at the individual and group levels via Principal Component Analysis (PCA). The dimensionally reduced data were next decomposed into ICs by means of ICA:

$$X = AS, \tag{1}$$

Where X denotes the *T-by-V* (time by voxel) group dimensionally reduced matrix of fMRI brain responses, A represents an unknown *N-by-N* mixing matrix, and S is an unknown *N-by-V* matrix containing the N ICs that are maximally independent. The rows and columns of S and A are the spatially independent images and the spatially independent time courses associated with those images. ICASSO, a robust analysis tool, was used to perform ICA by running the ICA algorithm iteratively and clustering similar IC estimates (18). The ICA algorithm used was FastICA (19), which has yielded consistent results for fMRI data analyses in the literature (20). We used a maximum of 100 different randomly initialized unmixing matrices up to convergence.

One caveat of the ICA approach is that the number of ICs into which the fMRI data is decomposed must be set a priori. This value, also known as ICA model order, represents the latent dimensionality of the data, and can thus have a significant impact on the spatial characteristics of the estimated functional networks and their parcellation into sub-networks (21). However, model order selection, i.e., inferring the appropriate number of ICs is a non-trivial problem still under investigation (22). To tackle this problem, we followed the procedure described in Burunat et al. (2017), which involved performing decomposition assuming ten different model orders. Next, the GICA3 back-reconstruction algorithm was used to reconstruct participant-specific IC spatial maps and associated temporal courses, whereby the mixing matrix A was back-projected to the PCA subject space (24). These participant-specific IC maps along with their respective temporal courses enabled statistical inferences to be drawn from group (musicians vs nonmusicians) comparisons.

The utilization of back projection for subject-level comparisons offers a robust and reliable method for estimating individual spatial maps and time courses. By deriving group-level ICA spatial maps from all subject time courses, we achieve improved parcellation compared to alternative approaches such as individual subject ICA followed by alignment (24). Moreover, the process mirrors that of dual regression, ensuring consistency and reliability in estimating subject-specific components. Despite potential information loss during PCA steps, which typically retains over 99.99% of variability, individual spatial maps and time courses remain effectively independent of each other, conditional on the shared spatial map. This methodology underscores the strength and validity of back projection for subject-level comparisons in our analysis.

GICA3: back-reconstruction of subject-specific IC spatial maps and temporal courses

Once the unmixing matrix has been estimated, subject-specific IC spatial maps and associated temporal courses can be reconstructed, enabling statistical inferences between groups, for which various multi-subject ICA approaches have been proposed. Among the existing methods, recent evidence suggests that GICA3 provides both the most robust results with the most intuitive interpretation derived from its mathematical properties, such as the aggregate IC spatial map being the sum of the back-reconstructed subject-specific spatial maps (24).

We applied GICA3 on the ICA results to obtain subject-level IC spatial maps through back-reconstruction. This allowed us to conduct a one-sample Wilcoxon signed rank test ($p < 0.001$) across the subject-level back-reconstructed spatial maps to assess the significance of the spatial maps associated with the time-shifted boundary regressor. Similarly, to test for group differences, non-parametric group comparisons (two-sample Wilcoxon signed rank tests, two-tailed, alpha = 0.05) were performed between groups.

Identification of IC temporal courses associated with boundary processing

In order to obtain the IC spatial maps associated with boundary processing, we determined the IC component that was best predicted by the time-shifted regressor for each phase of the transition

(before, during, and after the boundary) across all model orders. We used Spearman's rank correlation coefficient as a suitable non-parametric measure of statistical dependence. This choice was made because the potential relationship between the time-shifted boundary regressor and the IC-associated temporal courses was not necessarily expected to be linear.

The significance of the correlation coefficients had to be estimated due to the intrinsic serial correlation between adjacent fMRI samples, which reduces the effective degrees of freedom in the data. These were estimated by computing the cross correlation between IC time course and time-shifted boundary regressor (13). The estimate of effective degrees of freedom was averaged across model orders and subsequently used to compute the significance of the correlations by dividing the Fisher Z transformed correlation coefficients (27) by the standard error $1/\sqrt(df-3)$, where df represents the effective degrees of freedom. This approach has been applied in prior studies (refer to 25 and 26).

For each model order, the significance threshold was adjusted using the false discovery rate (FDR) criterion (q < 0.05). underwent correction for multiple comparisons using the false discovery rate (FDR) criterion (q < 0.05). The most significant IC (p < 0.001) was determined for each of the lags (before, during, and after the boundary transitions) across all model orders.

Once the IC time courses best predicted by the boundary regressor were identified (based on their highest correlation coefficient across all model orders), their associated IC spatial maps were identified. Subsequently, to assess the significance of these spatial maps, a statistical test was employed to generate maps indicating connectivity significantly different from zero. Specifically, a one-sample Wilcoxon signed rank test (alpha = 0.001) was employed for this purpose. This nonparametric test allowed for the comparison of spatial distributions of brain activity against a null hypothesis of zero activity for each lag (pre-, during, and post- boundary transitions). These IC maps defined relevant networks at the whole participant pool level.

Cluster correction for multiple comparisons

To account for multiple comparisons, a non-parametric cluster-wise correction approach was used (FWE = 0.05), whereby participant-level IC spatial maps were bootstrap resampled with replacement from the pool of back-projected IC maps within a given model order (i.e., for model order N, 36 IC spatial maps were randomly drawn from a total of 36∗N IC maps). The sample was then t-tested and thresholded (one-sample Wilcoxon signed rank test; p < 0.001). By running 5000 iterations, an empirical distribution of cluster sizes was generated per model order. Bootstrap resampling within a given model order ensures not only that the spatial maps are uncorrelated, but also that the spatial autocorrelation structure is consistent among them.

Granger causality analyses

We aimed to test the hypothesis that the two ICA networks involved in event boundary processing have directional or causal influence. Specifically, we predicted that the Early Auditory Network, active before the boundary transition, drove the engagement of the Boundary Transition Network, responsible for processing the boundary transition onwards.

GCA is used to measure the predictive value of one time series in forecasting the values of another. If incorporating past values of variable x improves the prediction of variable y's current value, we say that x Granger causes y. This technique relies on the concept of temporal precedence to identify the direction of causality based on the information present in the data.

We used the MVGC toolbox compute time-domain pairwise Granger causality (GC) estimates (28). The GCA was performed separately for each subject using back-reconstructed subject-level ICA temporal courses associated with both networks.

Model order estimation

In the context of GC analysis, model order estimation refers to the process of selecting the appropriate order (or lag length) of the autoregressive (AR) model used to estimate GC between

two or more time series variables. The AR model is a common tool used to model time series data, and it assumes that each observation in a time series is a linear combination of its past observations. The order of the AR model determines how many past observations are included in the model.

Determining the optimal model order for Granger causality analysis in fMRI data depends on the specific characteristics of the data and the research question of interest. In general, the optimal model order for fMRI data may vary depending on the specific dataset and analysis approach.

We determined the optimal model order for the Granger causality (GC) analysis using both the Akaike information criterion (AIC) and the Bayesian Information Criterion (BIC). These methods balance model fit and complexity to select the best trade-off between the two. Both the AIC and BIC yielded a model order of TR = 3 (6 seconds) as the optimal model order for GCA. This model order struck the right balance by effectively capturing the dynamic interactions among variables while avoiding overfitting. Therefore, Granger causality took into account a lag of 6 seconds into the past.

Difference of influence term

Spurious causal influences can arise due to the low temporal resolution and hemodynamic blurring in the fMRI signal, where an unidirectional influence can turn into a bidirectional interaction due to the loss of dynamic stochastic information in both channels of the system (29). To overcome this issue, a difference of influence term ($F_{x \to y} - F_{y \to x}$) was introduced into the GCA. This term allows to compare the forward influence of the first network on the second network and the reverse influence of the second network on the first. If the difference between these influences is statistically significant, it suggests a unidirectional causal influence from one network to the other. By considering this difference, redundant bidirectional connections in the network can be eliminated, enhancing the specificity of the inferred causal relationships.

The statistical significance of the difference was evaluated by generating a null distribution of difference of influence terms $F_{x \to y} - F_{y \to x} = 0$ on simulated data (5000 surrogate time series) with comparable temporal properties (by means of using Fourier resampling).

If the difference between the two influences is significant, it suggests a unidirectional causal influence less likely to be spurious than a bidirectional influence. Note that positive values of the influence difference term suggest a directional influence from X to Y, while negative values indicate influence in the reverse direction.

Figure S1. Dendrogram #1

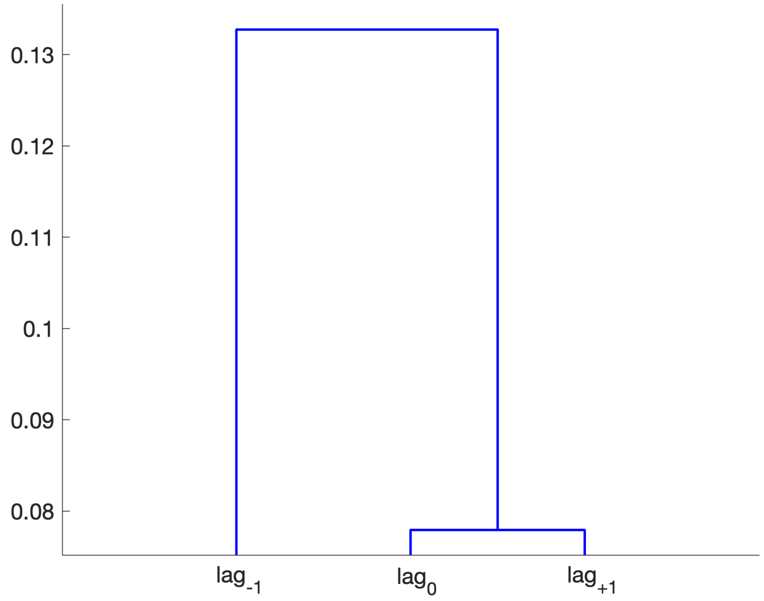

Figure S1. Dendrogram illustrating the spatial relationships among vectorized continuous z-score spatial maps corresponding to different lags. The clustering analysis, conducted using the cosine distance metric and Ward linkage method, exhibits a pattern akin to that observed in ICA analyses.

Figure S2. Dendrogram #2

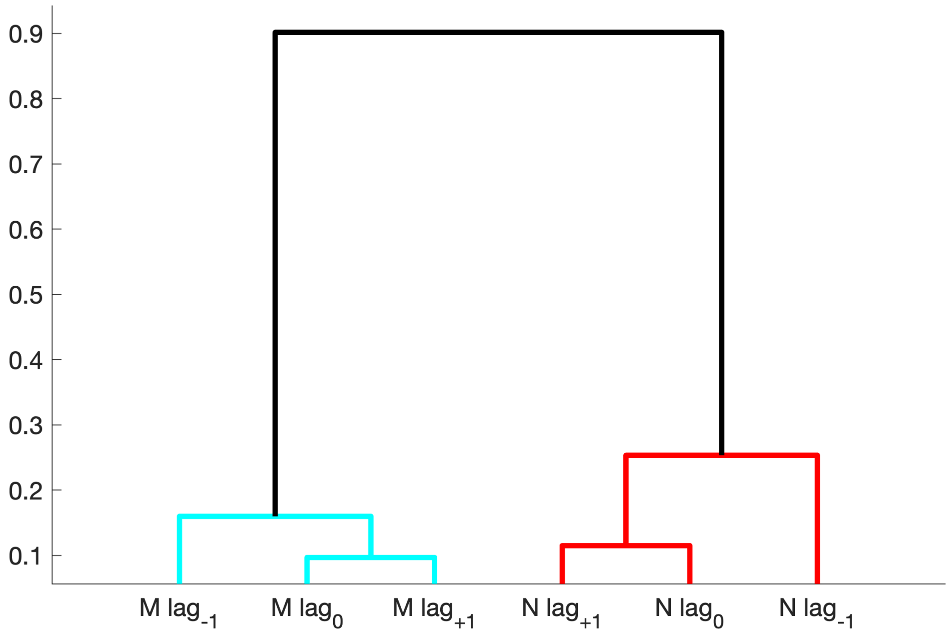

Figure S2. Dendrogram illustrating hierarchical clustering of group maps based on lag averages

for both musician and non-musician groups using an identical approach as in Figure S1. Notably, consistent clustering patterns emerge for lag 0 and lag +1 maps across both groups, distinct from lag -1, aligning with previous findings (refer to Figure S1; M: musicians; N: nonmusicians).

Abbreviations used in the Tables

Middle frontal gyrus	MFG
Superior frontal gyrus	SFG
Inferior temporal gyrus	ITG
Middle temporal gyrus	MTG
Superior temporal gyrus	STG
Middle Occipital Gyrus	MOG
Inferior Occipital Gyrus	IOG
Supramarginal gyrus	SMG
Parahippocampal gyrus	SHG
Inferior parietal, but supramarginal and angular gyri	Parietal Inf
Posterior cingulate and paracingulate gyrus	CGp and PCG
Median cingulate and paracingulate gyrus	CGm and PCG
Anterior cingulate and paracingulate gyrus	CGa and PCG
L / R	Left / Right

Table S1. Table related to Figure 2 (GLM results)

NEGATIVE						
**Left hemisphere**	k	x	y	z	Zval	BA
Cluster #1: N = 4367						
MFG (L)	924	-26	54	2	-5,91	11
SFG (L)	852	-24	54	2	-5,67	11
SFG, medial (L)	843	-12	60	20	-5,57	10
IFG, medial orbital (L)	414	-10	44	-10	-5,78	10
SFG, medial (R)	333	12	62	4	-5,74	10
IFG, medial orbital (R)	277	10	64	-2	-4,89	11
CGa and PCG	175	-6	46	-4	-5,42	10
SFG, orbital part (L)	132	-24	56	-2	-5,75	11
Gyrus rectus (R)	72	2	36	-16	-4,45	0
SFG (R)	62	14	66	0	-5,07	10
CGa and PCG (R)	49	2	52	8	-4,57	10
Gyrus rectus (L)	41	0	36	-16	-4,38	11
MFG, orbital part (L)	38	-44	56	-2	-4,24	46
SFG, orbital part (R)	11	14	66	-2	-4,49	11
Cluster #2: N = 1564						
Angular gyrus (L)	675	-46	-60	42	-6,03	39
Parietal Inf (L)	318	-48	-60	44	-6,11	39
MOG (L)	280	-50	-78	12	-5,07	19
MTG (L)	87	-48	-72	14	-4,69	37
Cluster #3: N = 955						
Precuneus (L)	610	-2	-58	42	-5,85	0
Precuneus (R)	205	2	-60	42	-5,42	0
CGp and PCG (L)	72	-6	-44	32	-4,81	23
CGm and PCG (L)	38	-8	-44	32	-4,65	23
Cluster #4: N = 626						
ITG (L)	357	-62	-24	-20	-5,84	20
MTG (L)	237	-60	-28	-16	-5,92	20
**Right hemisphere**						
Cluster #5: N = 1637						
Angular gyrus (R)	670	50	-56	30	-6,76	39
MTG (R)	315	54	-74	8	-7,2	19
MOG (R)	249	42	-72	24	-5,65	39
Parietal Inf (R)	214	58	-56	40	-6,53	39
ITG (R)	107	54	-72	-6	-6,76	0
IOG (R)	27	52	-76	-4	-5,48	19
SMG (R)	20	54	-46	42	-4,78	40
Cluster #6: N = 663						
SFG (R)	362	16	34	52	-6,42	9
MFG (R)	255	34	26	48	-4,98	9
SFG, medial (R)	32	16	34	58	-5,29	8
Cluster #7: N = 446						
ITG (R)	343	62	-26	-24	-5,63	20
MTG (R)	101	70	-24	-16	-5,77	21
Cluster #8: N = 219						
MFG (R)	135	34	54	12	-5,39	10
SFG (R)	72	34	54	10	-5,49	10
MFG, orbital part (R)	10	36	60	-2	-4,45	10
Cluster #9: N = 102						
Crus II of CER (R)	77	50	-66	-44	-5,26	0
Crus I of CER (R)	25	48	-74	-38	-4,2	0
Cluster #10: N = 92						
Fusiform gyrus (R)	60	30	-42	-14	-5,11	37
PHG (R)	31	30	-38	-12	-4,22	37

POSITIVE						
**Left hemisphere**	k	x	y	z	Zval	BA
Cluster #1: N = 5514						
STG (L)	2042	-62	-34	12	16,2	22
MTG (L)	1626	-52	-32	8	12,4	22
SMG (L)	300	-66	-24	14	8,3	22
Rolandic operculum (L)	182	-40	-34	14	13,5	41
Heschl's gyrus (L)	178	-36	-32	12	10,4	48
Temporal pole, STG (L)	137	-56	6	-12	7,19	38
Postcentral gyrus (L)	63	-64	-22	16	8,08	22
Insula (L)	29	-34	-32	20	7,78	48
Cluster #2: N = 793						
Crus II of CER (L)	262	-18	-78	-44	8,21	0
Crus I of CER (L)	153	-18	-76	-34	7,39	0
Lobule VI of CER (L)	126	-22	-72	-26	6,86	19
Lobule VIIB of CER (L)	123	-16	-76	-46	8,21	0
Lobule VIII of CER (L)	69	-18	-72	-40	6,87	0
Cluster #3: N = 458						
Precentral gyrus (L)	219	-50	-6	54	6,64	6
Postcentral gyrus (L)	194	-50	-8	54	6,31	0
Cluster #4: N = 346						
IFG, opercular part (L)						
IFG, triangular part (L)	104	-42	10	24	7,04	48
Precentral gyrus (L)	16	-40	8	30	5,15	44
Cluster #5: N = 326						
Insula (L)	168	-38	22	2	6,46	47
IFG, orbital part (L)	80	-36	24	-4	6,21	47
IFG, triangular part (L)	74	-38	24	0	6,33	47
**Right hemisphere**						
Cluster #6: N = 101712						
STG (R)	2753	58	-28	12	19,4	42
MTG (R)	1771	60	-40	10	18,6	42
IFG, triangular part (R)	986	50	18	24	9,41	48
IFG, opercular part (R)	777	52	18	26	9,19	48
Precentral gyrus (R)	768	50	4	44	8,21	6
Temporal pole, STG (R)	372	52	10	-16	8,21	38
SMG (R)	365	68	-26	18	13,2	22
Rolandic operculum (R)	363	46	-32	18	13	48
MFG (R)	258	48	-2	52	8,21	6
Insula (R)	231	40	26	-2	8,21	47
Heschl's gyrus (R)	215	50	-20	8	12,8	48
IFG, orbital part (R)	174	42	30	-4	8,21	47
Postcentral gyrus (R)	159	66	-16	14	9,3	0
Temporal pole, MTG (R)	79	52	4	-18	8,21	21
Cluster #7: N = 421						
SMA (R)	322	6	4	66	8,21	6
SMA (L)	98	0	2	66	6,59	0
Cluster #8: N = 279						
Vermis III of CER	32	4	-38	-8	6,89	30
Lingual gyrus (R)	11	12	-30	-6	5,41	27
Thalamus (R)	9	12	-28	-2	5,32	0

Activations and deactivations at 0 lag are displayed at $p < 0.001$, cluster-wise corrected, FWE = 0.05. clusters were obtained via the 18-connectivity scheme employed in SPM. Table reports within-cluster region size (k; i.e., number of voxels), peak Z-statistic value per region within the cluster with its respective MNI coordinates, and Brodmann area (BA). Voxels identified as white matter or voxels encroaching very small regions within the cluster (k< 5 voxels) were discarded from the resulting table.

Table S2 A. Table related to Figure 3 (ICA results; Early Auditory Network)

NEGATIVE						
Right hemisphere	k	x	y	z	Zval	BA
Cluster #1: N = 59						
Rolandic operculum (R)	52	56	-14	12	-4,25	48
Postcentral gyrus (R)	7	62	-14	16	-3,64	48
Cluster #2: N = 51						
MTG (R)	32	68	-24	-18	-4,22	21
ITG (R)	19	68	-24	-20	-4,16	20

POSITIVE						
Left hemisphere	k	x	y	z	Zval	BA
Cluster #1: N = 588						
STG (L)	389	-66	-46	18	5,18	22
MTG (L)	130	-60	-38	10	4,66	42
Right hemisphere						
Cluster #2: N = 961						
STG (R)	701	62	-42	12	5,22	42
MTG (R)	153	62	-40	10	5,22	42
Supramarginal gyrus (R)	97	66	-44	24	5,02	22

IC spatial map corresponding to the Early Auditory Network (one-sample Wilcoxon signed rank test, $p < 0.001$, cluster-wise corrected, FWE = 0.05). Table reports within-cluster region size (k; i.e., number of voxels), peak Z-statistic value per region within the cluster with its respective MNI coordinates, and Brodmann area (BA). Voxels identified as white matter or voxels encroaching very small regions within the cluster (k< 5 voxels) were discarded from the resulting table.

Table S2 B. Table related to Figure 3 (ICA results; Boundary Transition Network)

NEGATIVE

Left hemisphere	k	x	y	z	Zval	BA
Cluster #1: N = 934						
MTG (L)	194	-18	44	26	-5,18	32
STG, medial (L)	174	-10	56	12	-5,22	10
STG (L)	168	-22	34	30	-5,22	9
mOFC (L)	116	-12	36	-10	-4,8	11
STG, orbital part (L)	81	-18	52	-14	-5,15	11
CGa and PCG (L)	43	-14	42	-6	-4,69	10
MTG, orbital part (L)	29	-28	46	-6	-5,13	11
Gyrus rectus (L)	11	-10	36	-16	-4,44	11
Cluster #2: N = 615						
Crus II of CER (L)	212	-24	-74	-38	-5,18	0
Crus I of CER (L)	131	-20	-72	-38	-5,18	0
Lobule VIIB of CER (L)	124	-16	-70	-38	-5,15	0
Lobule VI of CER (L)	69	-16	-70	-26	-4,99	0
Lobule VIII of CER (L)	68	-20	-72	-40	-5,18	0
Cluster #3: N = 607						
MTG (L)	320	-44	-60	12	-5,11	37
Supramarginal gyrus (L)	36	-48	-46	28	-5,07	48
STG (L)	19	-46	-46	12	-4,42	21
Cluster #4: N = 570						
ITG (L)	371	-50	-12	-40	-5,22	20
MTG (L)	152	-56	-28	-16	-5,18	20
Cluster #5: N = 520						
MOG (L)	147	-40	-86	14	-5,22	19
Parietal Inf (L)	104	-52	-44	42	-5,08	40
Angular gyrus (L)	80	-36	-56	32	-5,1	39
MTG (L)	79	-48	-68	16	-5,22	39
Cluster #6: N = 240						
IFG, opercular part (L)	158	-32	12	30	-5,22	44
IFG, triangular part (L)	60	-34	10	24	-5	48
Precentral gyrus (L)	13	-32	8	30	-5,05	44
Cluster #7: N = 175						
Insula (L)	105	-26	22	4	-5,15	48
IFG, triangular part (L)	38	-30	32	0	-5,07	47
IFG, orbital part (L)	28	-40	32	-4	-4,5	47
Cluster #8: N = 162						
Precuneus (L)	117	-6	-60	56	-5,19	7
CGp and PCG (L)	21	-6	-42	30	-4,47	23
CGm and PCG (L)	20	-14	-50	34	-4,27	0
	20	-14	-50	34	-4,27	0
Cluster #9: N = 121						
MOG (L)	104	-40	-78	28	-5,22	39
Angular gyrus (L)	10	-44	-76	30	-5	39
Cluster #10: N = 84						
STG (L)	43	-20	18	54	-5,11	8
MTG (L)	41	-24	14	56	-5,02	8
Cluster #11: N = 60						
IFG, triangular part (L)	60	-56	30	18	-4,74	45

Right hemisphere	k	x	y	z	Zval	BA
Cluster #12: N = 1978						
IFG, triangular part (R)	665	32	16	24	-5,22	48
IFG, opercular part (R)	507	32	6	28	-5,21	48
Precentral gyrus (R)	302	32	4	30	-5,21	44
Insula (R)	114	30	28	6	-5,04	48
IFG, orbital part (R)	111	32	32	-6	-4,91	47
MTG (R)	47	34	20	22	-5,21	48
Postcentral gyrus (R)	5	52	-2	32	-3,92	6
Cluster #13: N = 1357						
MTG (R)	591	50	-44	2	-5,22	21
STG (R)	247	54	-48	20	-5,22	42
Supramarginal gyrus (R)	91	48	-42	26	-5,22	48
Temporal pole, MTG (R)	9	50	6	-24	-4,64	21
Cluster #14: N = 1119						
MTG (R)	319	48	-76	14	-5,22	19
MOG (R)	312	48	-78	12	-5,22	19
Angular gyrus (R)	279	46	-64	24	-5,22	39
ITG (R)	114	46	-68	-10	-5,22	37
IOG (R)	27	46	-76	-6	-5,11	19
Parietal Inf (R)	26	48	-44	38	-4,78	40
Supramarginal gyrus (R)	10	48	-46	36	-5,02	48
Cluster #15: N = 348						
ITG (R)	316	56	-28	-22	-5,22	20
MTG (R)	25	60	-14	-24	-4,47	20
Cluster #16: N = 243						
mOFC (R)	110	12	40	-14	-4,97	11
Gyrus rectus (R)	60	14	38	-16	-5,15	11
CGa and PCG (R)	53	10	34	2	-5,13	11
Gyrus rectus (L)	12	2	26	-18	-4,34	11
Cluster #17: N = 211						
Parahippocampal gyrus (R)	100	34	-22	-24	-5,04	20
Fusiform gyrus (R)	89	36	-34	-26	-5,15	20
Cluster #18: N = 178						
STG, medial (R)	138	14	50	18	-5,21	32
CGa and PCG (R)	20	14	48	20	-5,11	32
STG (R)	19	16	58	2	-5,15	10
Cluster #19: N = 96						
STG (R)	57	22	16	56	-4,78	8
MTG (R)	38	28	20	46	-4,41	8

POSITIVE

Left hemisphere	k	x	y	z	Zval	BA
Cluster #1: N = 2540						
STG (L)	1580	-54	-14	-2	5,22	48
MTG (L)	377	-50	-20	0	5,22	48
Heschl's gyrus (L)	156	-48	-16	6	5,22	48
Rolandic operculum (L)	111	-38	-30	12	5,22	48
Temporal pole, STG (L)	87	-58	6	-4	4,72	38
Supramarginal gyrus (L)	64	-64	-24	14	5,13	42
Postcentral gyrus (L)	34	-64	-22	14	5,08	22

Right hemisphere	k	x	y	z	Zval	BA
Cluster #2: N = 2794						
STG (R)	1796	52	-2	-10	5,22	21
MTG (R)	227	66	-22	-6	5,22	21
Temporal pole, STG (R)	215	58	6	-8	5,22	38
Rolandic operculum (R)	193	62	-8	8	5,22	22
Heschl's gyrus (R)	189	48	-16	4	5,22	48
Supramarginal gyrus (R)	92	56	-28	18	5,22	42
Insula (R)	36	46	-12	4	5,22	48
Postcentral gyrus (R)	17	66	-16	14	5	0

IC spatial map corresponding to the Boundary Transition Network (one-sample Wilcoxon signed rank test, $p < 0.001$, cluster-wise corrected, FWE = 0.05). Table reports within-cluster region size (k; i.e., number of voxels), peak Z-statistic value per region within the cluster with its respective MNI coordinates, and Brodmann area (BA). Voxels identified as white matter or voxels encroaching very small regions within the cluster (k< 5 voxels) were discarded from the resulting table.

Table S3 A. Table related to Figure 5 (group comparison; Early Auditory Network)

MUSICIANS>NMUSICIANS						
Left hemisphere	k	x	y	z	Zval	BA
Cluster #1: N = 138						
MTG (L)	138	-56	-46	4	2,64	22

NONMUS>MUSICIANS						
Left hemisphere	k	x	y	z	Zval	BA
Cluster #1: N = 188						
STG (L)	27	-42	-24	2	-2,67	48
Heschl's gyrus (L)	16	-32	-28	6	-2,39	48
MTG (L)	6	-44	-24	0	-2,42	48
Right hemisphere						
Cluster #2: N = 164						
IFG, triangular part (R)	71	42	20	22	-2,48	48
IFG, opercular part (R)	25	42	14	14	-2,45	48
Cluster #3: N = 155						
MTG (R)	34	48	-6	-16	-2,33	21
STG (R)	19	48	-6	-14	-2,29	21
Cluster #4: N = 107						
MOG (R)	92	42	-74	28	-3,05	39
Angular gyrus (R)	15	44	-70	30	-2,1	39
Cluster #5: N = 101						
Heschl's gyrus (R)	15	40	-26	6	-1,88	48
STG (R)	15	46	-30	8	-2,17	48
Insula (R)	13	36	-22	2	-2,77	48

Group comparison map resulting from the Early Auditory Network (two-sample Wilcoxon signed rank tests, $p < 0.05$, cluster-wise corrected, FWE = 0.05). Table report within-cluster region size (k; i.e., number of voxels), peak Z-statistic value per region within the cluster with its respective MNI coordinates, and Brodmann area (BA). Voxels identified as white matter or voxels encroaching very small regions within the cluster (k< 5 voxels) were discarded from the resulting table.

Table S3 A. Table related to Figure 5 (group comparison; Boundary Transition Network)

MUSICIANS>NMUSICIANS						
Left hemisphere	k	x	y	z	Zval	BA
Cluster #1: N = 108						
STG (L)	57	-60	-30	16	2,55	42
Rolandic operculum (L)	25	-42	-26	14	2,39	48
Heschl's gyrus (L)	21	-38	-26	12	2,83	48
Right hemisphere						
Cluster #2: N = 93						
IFG, triangular part (R)	75	56	38	0	3,05	0
IFG, orbital part (R)	18	48	34	-4	2,67	47
Cluster #3: N = 80						
STG (R)	58	48	-38	10	2,29	41
MTG (R)	9	48	-46	16	1,95	41
Cluster #4: N = 72						
IFG, opercular part (R)	61	60	14	32	3,24	0
Precentral gyrus (R)	5	60	14	34	2,67	0

NONMUS>MUSICIANS						
Left hemisphere	k	x	y	z	Zval	BA
Cluster #1: N = 172						
STG (L)	51	-38	-38	16	-2,61	41
Supramarginal gyrus (L)	26	-50	-46	26	-2,52	48
Rolandic operculum (L)	15	-38	-36	16	-2,23	48
Heschl's gyrus (L)	13	-32	-30	14	-2,17	48
Right hemisphere						
Cluster #2: N = 656						
SFG, medial (R)	199	4	58	2	-3,15	10
SFG, medial (L)	185	0	58	2	-2,71	0
mOFC (L)	101	0	54	-2	-2,67	0
CGa and PCG (L)	65	-4	44	-4	-2,55	10
mOFC (R)	58	6	60	-2	-2,93	0
CGa and PCG (R)	33	6	36	-6	-2,2	11
Cluster #3: N = 119						
Angular gyrus (R)	106	48	-66	30	-2,99	39
MOG (R)	13	48	-66	28	-2,55	39

Group comparison map resulting from the Boundary Transition Network (two-sample Wilcoxon signed rank tests, $p < 0.05$, cluster-wise corrected, FWE = 0.05). Table report within-cluster region size (k; i.e., number of voxels), peak Z-statistic value per region within the cluster with its respective MNI coordinates, and Brodmann area (BA). Voxels identified as white matter or voxels encroaching very small regions within the cluster (k< 5 voxels) were discarded from the resulting table.

Legends for Movie S1 (separate file). Dynamic brain changes during boundary transitions.

Movie S1 exhibits the engagement of the Early Auditory Network and Boundary Transition Network, highlighting the dynamic shifts in functional connectivity and adaptive responses of the two networks during the processing of musical boundaries (t = -1, 0, and +1, representing moments before, during, and after the boundary transition (t = TR = 2s). The smooth transition between these two distinct functional networks was achieved through the utilization of 3D linear interpolation for visualizing purposes. Three musical excerpts representing boundary transition peaks were selected per musical stimuli to highlight the dynamic shifts in functional brain networks.

SI References

1. B. P. Gold, M. J. Frank, B. Bogert, E. Brattico, Pleasurable music affects reinforcement learning according to the listener. *Frontiers in Psychology* **4**, 1–19 (2013).
2. V. Alluri, *et al.*, Musical expertise modulates functional connectivity of limbic regions during continuous music listening. *Psychomusicology* **25**, 443–454 (2015).
3. I. Burunat, *et al.*, Action in Perception: Prominent Visuo-Motor Functional Symmetry in Musicians during Music Listening. *Plos One* **10**, e0138238 (2015).
4. P. Saari, I. Burunat, E. Brattico, P. Toiviainen, Decoding Musical Training from Dynamic Processing of Musical Features in the Brain. *Scientific Reports* **8**, 708 (2018).
5. J. Ashburner, K. J. Friston, Voxel-based morphometry--the methods. *NeuroImage* **11**, 805–21 (2000).
6. A. Evans, M. Kamber, D. Collins, D. MacDonald, "An MRI-based probabilistic atlas of neuroanatomy" in *Magnetic Resonance Scanning and Epilepsy*, S. D. Shorvon, Ed. (New York: Plenum Press, 1994), pp. 263–274.
7. D. Zicarelli, M. Puckett, Max/MSP (2002). http://www.cycling74.com/ (accessed 17 March 2024).
8. B. Silverman, *Density estimation for statistics and data analysis* (Chapman and Hall, 1986) (May 17, 2012).
9. M. Hartmann, O. Lartillot, P. Toiviainen, Multi-scale Modelling of Segmentation: Effect of Music Training and Experimental Task. *Music Perception* **34**, 192–217 (2016).
10. F. Beissner, A. Schumann, F. Brunn, D. Eisenträger, K.-J. Bär, Advances in functional magnetic resonance imaging of the human brainstem. *NeuroImage* **86**, 91–98 (2014).
11. E. Formisano, F. Esposito, F. Di Salle, R. Goebel, Cortex-based independent component analysis of fMRI time series. *Magnetic Resonance Imaging* **22**, 1493–1504 (2004).
12. W. S. Sohn, K. Yoo, Y. Jeong, Independent component analysis of localized resting-state functional magnetic resonance imaging reveals specific motor subnetworks. *Brain connectivity* **2**, 218–24 (2012).
13. B. J. Pyper, R. M. Peterman, Comparison of methods to account for autocorrelation in correlation analyses of fish data. *Canadian Journal of Fisheries and Aquatic Sciences* **2140**, 2127–2140 (1998).
14. W. Ebisuzaki, A Method to Estimate the Statistical Significance of a Correlation When the Data Are Serially Correlated. *J. Climate* **10**, 2147–2153 (1997).
15. M. J. McKeown, T. J. Sejnowski, Independent component analysis of fMRI data: examining the assumptions. *Human brain mapping* **6**, 368–72 (1998).

16. M. a. Lindquist, The Statistical Analysis of fMRI Data. *Statistical Science* **23**, 439–464 (2008).

17. J. S. Damoiseaux, *et al.*, Consistent resting-state networks across healthy subjects. *Proceedings of the national academy of sciences* **103**, 13848–13853 (2006).

18. J. Himberg, A. Hyvärinen, F. Esposito, Validating the independent components of neuroimaging time series via clustering and visualization. *NeuroImage* **22**, 1214–1222 (2004).

19. A. Hyvärinen, J. Karhunen, E. Oja, *Independent Component Analysis* (2001).

20. N. Correa, T. Adali, V. D. Calhoun, Performance of Blind Source Separation Algorithms for FMRI Analysis using a Group ICA Method. *Magnetic resonance imaging* **25**, 684–694 (2007).

21. A. Abou-Elseoud, *et al.*, Group-ICA Model Order Highlights Patterns of Functional Brain Connectivity. *Frontiers in Systems Neuroscience* **5**, 37 (2011).

22. A. Abou-Elseoud, *et al.*, The effect of model order selection in group PICA. *Human Brain Mapping* **31**, 1207–1216 (2010).

23. I. Burunat, V. Tsatsishvili, E. Brattico, P. Toiviainen, Coupling of Action-Perception Brain Networks during Musical Pulse Processing: Evidence from Region-of-Interest-Based Independent Component Analysis. *Frontiers in Human Neuroscience* **11** (2017).

24. E. B. Erhardt, *et al.*, Comparison of multi-subject ICA methods for analysis of fMRI data. *Human Brain Mapping* **32**, 2075–2095 (2011).

25. V. Alluri, *et al.*, Large-scale brain networks emerge from dynamic processing of musical timbre, key and rhythm. *NeuroImage* **59**, 3677–3689 (2012).

26. I. Burunat, *et al.*, The reliability of continuous brain responses during naturalistic listening to music. *NeuroImage* **124**, 224–231 (2016).

27. R. A. Fisher, *Statistical Methods for Research Workers, 11th edn.* (Oliver and Boy, London, 1950).

28. L. Barnett, A. K. Seth, The MVGC multivariate Granger causality toolbox: A new approach to Granger-causal inference. *Journal of Neuroscience Methods* **223**, 50–68 (2014).

29. W. W. S. Wei, "Time Series Analysis" in *The Oxford Handbook of Quantitative Methods in Psychology: Vol. 2: Statistical Analysis*, T. D. Little, Ed. (Oxford University Press, 2013), p. 0.
